# A co-expressed gene status of adenylate kinase 1/4 reveals prognostic gene signature associated with prognosis and sensitivity to EGFR targeted therapy in lung adenocarcinoma

**DOI:** 10.1038/s41598-019-48243-9

**Published:** 2019-08-23

**Authors:** Yi-Hua Jan, Tsung-Ching Lai, Chih-Jen Yang, Ming-Shyan Huang, Michael Hsiao

**Affiliations:** 1 Genomics Research Center, Academia Sinica, Taipei, Taiwan; 2 Department of Internal Medicine, Kaohsiung Municipal Ta-Tung Hospital, Kaohsiung Medical University, Kaohsiung, Taiwan; 3 Faculty of Medicine, College of Medicine, Kaohsiung Medical University, Kaohsiung, Taiwan; 4 Department of Internal Medicine, E-DA Cancer Hospital, School of Medicine, I-Shou University, Kaohsiung, Taiwan; 5 Department of Biochemistry, College of Medicine, Kaohsiung Medical University, Kaohsiung, Taiwan

**Keywords:** Prognostic markers, Non-small-cell lung cancer

## Abstract

Cancer cells utilize altered bioenergetics to fuel uncontrolled proliferation and progression. At the core of bioenergetics, adenine nucleotides are the building blocks for nucleotide synthesis, energy transfer and diverse metabolic processes. Adenylate kinases (AK) are ubiquitous phosphotransferases that catalyze the conversion of adenine nucleotides and regulate the homeostasis of nucleotide ratios within cellular compartments. Recently, different isoforms of AK have been shown to induce metabolic reprograming in cancer and were identified as biomarkers for predicting disease progression. Here we aim to systemically analyze the impact of all AK-associated gene signatures on lung adenocarcinoma patient survival and decipher the value for therapeutic interventions. By analyzing TCGA Lung Adenocarcinoma (LUAD) RNA Seq data, we found gene signatures from AK4 and AK1 have higher percentage of prognostic genes compared to other AK-gene signatures. A 118-gene signature was identified from consensus gene expression in AK1 and AK4 prognostic gene signatures. Immunohistochemistry (IHC) analyses in 140 lung adenocarcinoma patients showed overexpression of AK4 significantly correlated with worse overall survival (*P* = 0.001) whereas overexpression of AK1 significantly associated with good prognosis (*P* = 0.009). Furthermore, reduced AK4 expression by shRNA reduced the EGFR protein expression in EGFR mutation cells. The inhibition of AK4-AK1 signal might provide a potential target for synergistic effect in target therapy in lung cancer patients.

## Introduction

Non-small cell lung cancer (NSCLC) remains one of the most deadly types of lung cancer around the world^1^. Lung adenocarcinoma is the most subtype in NSCLC Patients with NSCLC are often diagnosed at advanced stage and the prognosis is usually dismal. Despite improvements in therapeutic modalities, the recurrence rate among NSCLC patients after first treatment is about 40% within 5 years^2^. Moreover, several driver mutations on well-defined oncogenes are associated with NSCLC tumorigenesis such as *EGFR*, *KRAS*, and *ALK*^3–5^. Since 2005, EGFR tyrosine kinase inhibitor (TKI) was used as targeted therapy agent for the treatment of NSCLC. However, in clinical trials, only a small percentage (9–26%) of patients with advanced NSCLC showed objective responses^6,7^. Subsequent studies have shown tumors harbor activating somatic mutations in EGFR are responsive to gefitinib or erlotinib treatment. However, resistance to EGFR TKI, such as secondary T790M mutation in EGFR, eventually occurs in most of the patients^8,9^. Some meta-analysis studies reported that EGFR-TKI significantly improves progression-free survival in patients with EGFR mutations but has no beneficial effect on overall survival in patients with EGFR mutations or wild-type EGFR^10,11^. Therefore, it is urgent to identify predictive biomarkers or gene signatures to assist therapeutic decisions.

Adenylate kinases (AK) are abundant phosphotransferases that transfer a phosphate from one ATP/GTP to one AMP and generate two ADP. So far, there are nine AK isoenzymes discovered in human tissues and they collectively regulate the adenine nucleotide composition within different cellular compartments to maintain cellular energy homeostasis^12^. AK1, along with AK5, AK7, AK8, and AK9, is a cytoplasmic AK, AK2 can be found in the mitochondrial intermembrane space, AK3 and AK4 are located at mitochondrial matrix, AK6 and AK9 are expressed in the nucleus. Until now, the link between AK isoforms expression and cancer development is still not fully understood. The cytosolic AK1 is highly expressed in heart, brain, and skeletal muscle. However, an additional gene product of AK1, AK1β, was shown to be a p53-inducible membrane-bound AK that regulates cell cycle arrest and therefore AK1 was often downregulated during tumorigenesis^13–15^. AK4 has been reported to be a stress-responsive protein for cell surviving when it bind to mitochondrial ADP/ATP translocase^16,17^. It was also identified as lung cancer progression marker that predicts poor survival and metastatic phenotype^18^. Moreover, AK4 was reported to involve in hypoxia adaptation and anti-cancer drug resistance by modulating mitochondrial bioenergetics^19^. The nucleus isoform AK6 was identified as a human coilin-interacting nuclear ATPase protein hCINAP that regulates ribosomal RNA biogenesis and it was reported to be essential for embryogenesis, tumor growth, and self-renewal of cancer stem cells^20,21^.

Here we aimed to systematically investigate the association between gene expression signatures of different AK isoforms and lung adenocarcinoma patient outcomes using TCGA LUAD RNA Seq data set. We identified gene expression signatures that co-expressed with AK1 and AK4 have high percentage of prognostic genes as evaluated by PRECOG prognostic z-scores^22^. A consensus 118-gene signature of AK1 and AK4 was identified as a co-expressed gene set that is significantly associated the prognosis of lung adenocarcinoma patients. We also found this gene signature is significantly enriched in genes downregulated by irreversible EGFR inhibitor treatment in Gefitinib-resistant cells. Moreover, through pathway analysis and upstream regulator analyses, we also identified putative metabolic pathways and transcription factor networks that potentially mediate lung adenocarcinoma patient outcomes. Finally, by correlating the status of AK4 mRNA expression with drug sensitivity data in lung adenocarcinoma cell lines, we proposed lung cancer cells that have high levels of AK4 might be sensitive to EGFR target therapy.

## Materials and Methods

### Specimens

A total of 140 lung adenocarcinoma samples were collected at Kaohsiung Medical University Hospital with the Institutional Review Boards (IRB) approval and permission from the ethics committees of Kaohsiung Medical University Hospital (KMUHIRB-E(I)-20160099). IRB of Kaohsiung Medical University Hospital also approved a waiver of informed consent as all data were analyzed anonymously, and no identifying information related to the participants was included. All studies were carried out in accordance with the regulations and guidelines for the collection and use of human specimens for research at Kaohsiung Medical University Hospital. All patients were treated according to the standard treatment protocol. For patients with stage I, tumors were resected with no adjuvant chemotherapy. Patients with stage II-III were treated with platinum-based chemotherapy after tumor resection. Patients with non-operable locally invasive or metastatic disease were treated with chemotherapy with or without radiotherapy. The histologic types of lung cancer were diagnosed according to the World Health Organization (WHO) 2004 classification guideline. The pathological diagnosis of lung cancer were determined by the American Joint Committee on Cancer (AJCC) TNM classification. Overall survival time was defined as the period of time between first treatment and patient death, while disease-free survival time was defined as the interval after first treatment to disease relapse or death. The longes follow-up time was up to 200 months.

### Tissue microarray and immunohistochemical staining

Tissue cores from each specimen picked according to original hematoxylin and eosin slides and diagnostic information. IHC staining was performed using an automatic immunostainer (Discovery XT autostainer, Ventana, USA). Paraffin sections were dewaxed in a 60 °C oven, deparaffinized in xylene, and then rehydrated in graded EtOH. Antigen retrieval was performed using heat-induced TRIS-EDTA buffer for 30 minutes. Immunoreactivity of protein expressions was developed using 3, 3′-diaminobenzidine (DAB) peroxidase substrate kit (Ventana, USA). The slides were counterstained with hematoxylin. The antibodies were used to determine AK4 and AK1 expression: AK4 (Genetex, 1:200), and AK1 (Genetex, 1:100).

### IHC staining results assessment

The IHC staining results were evaluated and scored by two pathologists who were blinded to patient’s information. Protein expression of AK4 and AK1 in tumor cells were defined as: 0: no staining; 1: weak staining; 2: moderate staining; 3: strong staining. For AK1 scoring, no cytoplasmic staining or cytoplasmic staining less than 10% of tumor cells was defined as negative; weak staining or cytoplasmic staining more than 10% of tumor cells was defined as 1; moderate cytoplasmic staining in more than 10% of tumor cells was defined as 2; strong cytoplasmic staining in more than 10% of tumor cells was defined as 3. Score 0 and Score 1 were defined as low expression, whereas score 2 and score 3 were defined as high expression. For AK4 scoring, no mitochondrial staining or mitochondrial staining less than 10% of tumor cells was defined as negative; weak staining or mitochondrial staining more than 10% of tumor cells was defined as 1; moderate mitochondrial staining in more than 10% of tumor cells was defined as 2; strong mitochondrial staining in more than 10% of tumor cells was defined as 3. Score 0 and Score 1 were defined as low expression, whereas score 2 and score 3 were defined as high expression.

### The Cancer Genome Atlas gene expression data analysis

The Cancer Genome Atlas (TCGA) mRNA expression (RNASeq V2 RSEM) data for lung adenocarcinoma (LUAD provisional) were downloaded from the cBioPortal^23,24^. Coexpression analysis was used to identify gene signatures that associated with AK expression. AK-associated gene signatures were identified by selecting genes with the Pearson correlation coefficient more than 0.3 or less than −0.3.

The prognostic values for each gene in AK-associated signature was determined by querying PREdiction of Clinical Outcomes from Genomic Profiles (PRECOG) database^22^. Genes with survival Z-score more than 2 or less than −2 were defined as prognostic gene.

The expression profiles for each gene in normal and tumor tissue was determined by TPM (log_2_X + 1) from GEPIA (Gene Expression Profiling Interactive Analysis)^25^. Gene set enrichment analysis (GSEA) was analyzed as previous report^26^.

The activity of upstream factors in AK4 and AK1 gene signature were predicted by IPA Analysis (Ingenuity Systems) and the z-scores indicated the overall activation state of regulators (<0: inhibited, >0: activated). Z-score more than 2 or less than −2 were considered significant changes.

### Western blot analysis

Total protein lysate was harvested from variant culture cell lines with lysis buffer contains proteinase inhibitors on ice to detect endogenous proteins. After centrifugation, 40 μg protein lysate in loading buffer was loaded each lane. Proteins were separated and transferred by standard procedures for western blotting. After blocking and incubation with primary antibody, AK1 1:1000 (Genetex); AK4 1:2000 (Genetex); pEGFR 1:2000 (Cell Signaling); EGFR 1:2000 (SC-03, Santa Cruz); β-actin 1:10000 (Sigma), the luminescent signals were detected with ECL Pro reagent (Perkinelmer) after HRP-antibody hybridization. The images were recorded by LAS-3000 Imaging System (Fuji).

### Statistical analysis

Statistics analysis was performed on Excel or SPSS 17.0 software. Survival rates were calculated by the Kaplan-Meier method. Among all data, P-value < 0.05 was considered statistically significant. We applied two-tailed and unpaired Student’s t-tests for each pair sample comparison.

## Results

### Systemic analyses of adenylate kinase (AK) gene signatures identify a co-expressed gene set of AK4 and AK1 that impacts patient survival

**Figure 1:**
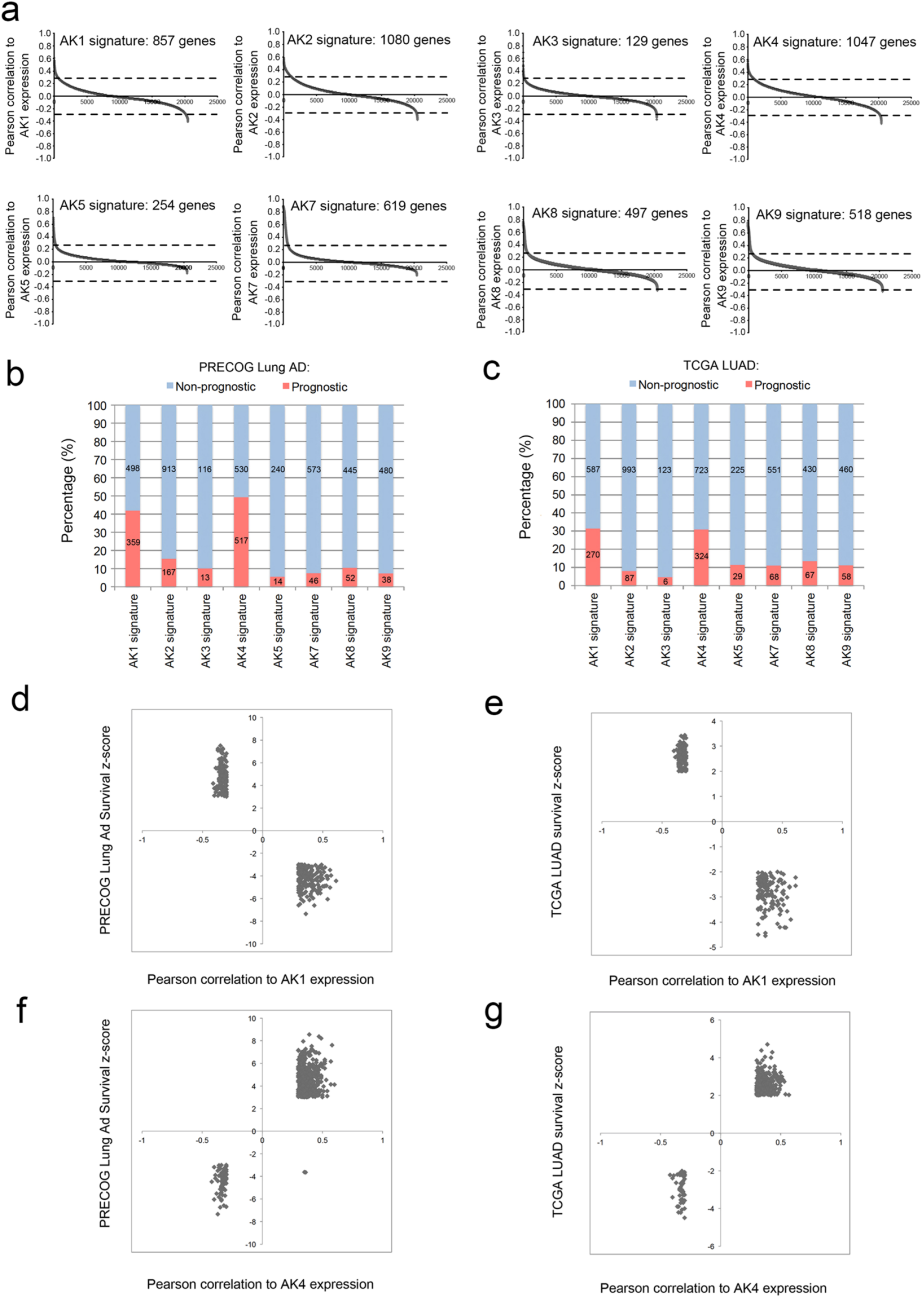
Identification of AK gene signatures in lung adenocarcinomas. (**a**) Identification of AK-signature in TCGA Lung adenocarcinoma (LUAD) dataset (n = 521). AK-associated genes were identified by correlating AK expression with the rest of the coding genes in the dataset and ranked according to Pearson correlation coefficient. AK gene signatures were defined by ±0.3 cutoff of Pearson correlation coefficient. Percentage of prognostic genes in each AK-signature queried by PRECOG (**b**) and/or TCGA (**c**) survival Z-score. Genes with survival Z-score more than 2 or less than -2 were defined as prognostic genes. Number in the bar chart indicates the numbers of gene as prognostic or non-prognostic genes in AK-signature. XY plot distribution of PRECOG Lung Ad prognostic genes (**d**) and TCGA LUAD prognostic genes (**e**) in AK1 gene signature. XY plot distribution of PRECOG Lung Ad prognostic genes (**f**) and TCGA LUAD prognostic genes (**g**) in AK4 gene signature.

We previously reported that adenylate kinase 4 (AK4) can be used as a lung cancer progression marker that enhances the invasion ability of lung cancer cells and may represent a biomarker of metastasis^18^. However, the interplay among AK isoforms and their impact on lung cancer pathogenesis remains unclear. To investigate the molecular profiles of each AK-associated gene signatures, we performed co-expression analysis individually for AK1, AK2, AK3, AK4, AK5, AK7, AK8, and AK9 using TCGA lung adenocarcinoma (LUAD) dataset and identified unique signatures for each AK (Fig. 1a and Supplementary Table S1). To evaluate the impact of AK-signatures on patient survival, we curated each gene in AK-signatures by querying PREdiction of Clinical Outcomes from Genomic Profiles (PRECOG) and TCGA survival Z-score^22^. By this mean, we could determine the percentage of prognostic genes in each AK signature. Notably, AK4- and AK1-gene signature have significant higher percentage of prognostic genes compared to the rest of the AK-signatures (Fig. 1b). Interestingly, genes that positively correlated with AK1 expression show better outcome while those negatively correlated with AK1 predict poor survival (Fig. 1d,e and Supplementary Table S2). On the other hand, genes that positively correlated with AK4 expression predict poor survival while those negatively correlated with AK4 predict better outcomes (Fig. 1f,g and Supplementary Table S2).

### Prognostic value of AK4 and AK1 expressions in lung adenocarcinoma patients

**Figure 2:**
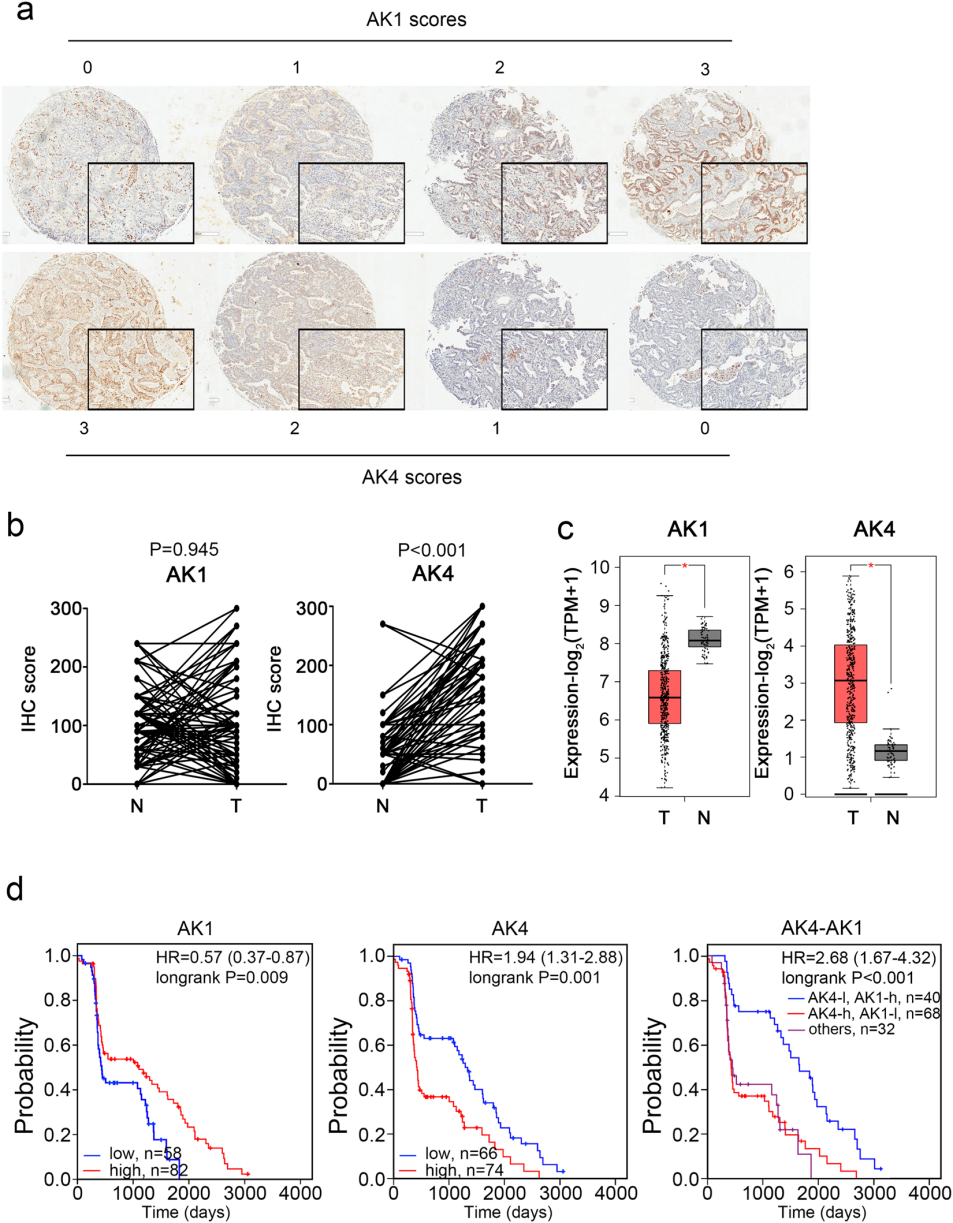
Kaplan-Meier survival analyses of AK4 and AK1 expression in lung adenocarcinoma patients. (**a**) Representative IHC staining and scoring of AK4 and AK1 protein expression. (**b**) IHC score of AK1 and AK4 in 123 N-T pair samples of lung adenocarcinoma. (**c**) The distribution of RNA expression of AK1 and AK4 in TCGA lung adenocarcinoma normal samples and tumor samples. (**d**) Kaplan-Meier analysis of overall survival and disease-free survival for AK4, AK1 and AK4-AK1 protein expression in 140 lung adenocarcinoma patients.

Among all 140 patients with lung adenocarcinoma, low cytoplasmic AK1 IHC immunoreactivity (score 0 and score 1) was observed in 58 (41.4%) cases while strong cytoplasmic AK1 IHC immunoreactivity (score 2 and score 3) was detected in 82 (58.6%) cases (Fig. 2a, upper panel). On the other hand, we identified 74 (52.9%) patients with high mitochondrial AK4 IHC immunoreactivity (score 2 and score 3) and 66 (47.1%) patients with low mitochondrial AK4 IHC immunoreactivity (Fig. 2a lower panel). In the comparison of normal and tumor part in lung cancer samples, AK4 usually overexpressed in tumor part (Fig. 2b, *P* < 0.001). Otherwise, AK1 expression have no significant differences in our clinical cohort. However, the opposite correlation of AK1 and AK4 was obviously in the TCGA lung adenocarcinoma cohort (Fig. 2c, both *P* < 0.001). Using Kaplan-Meier survival analysis, we found patients with high AK1 expression were significantly associated with better overall survival compared to those with low AK1 expression (Fig. 2d, left panel; *P* = 0.009). On the contrary, patients with high AK4 expression showed worse overall survival compared to those with low AK4 expression (Fig. 2d, central panel; *P* = 0.001).

Furthermore, when we combined the expression of AK4 and AK1 to analyze the prognostic impact. The predicted power of prognosis with combination subgrouping was better than AK4/AK1 alone (Fig. 2d). The hazard ratio in AK4-high and AK1-low group has highest risk (HR = 2.68, *P* < 0.001) to the AK4-low and AK1-high group. Not only in protein level, several other public lung cancer cohorts (TCGA LUAD, Dhananjay, Kerby, Lung meta-base, Kohno, and Rousseaux cohort, with gene expression profiles also presented similar trends (Supplementary Fig. S1)^27–32^.

### Identification of AK4 and AK1 consensus gene signature that predicts clinical outcomes and the response to EGFR inhibition

**Figure 3:**
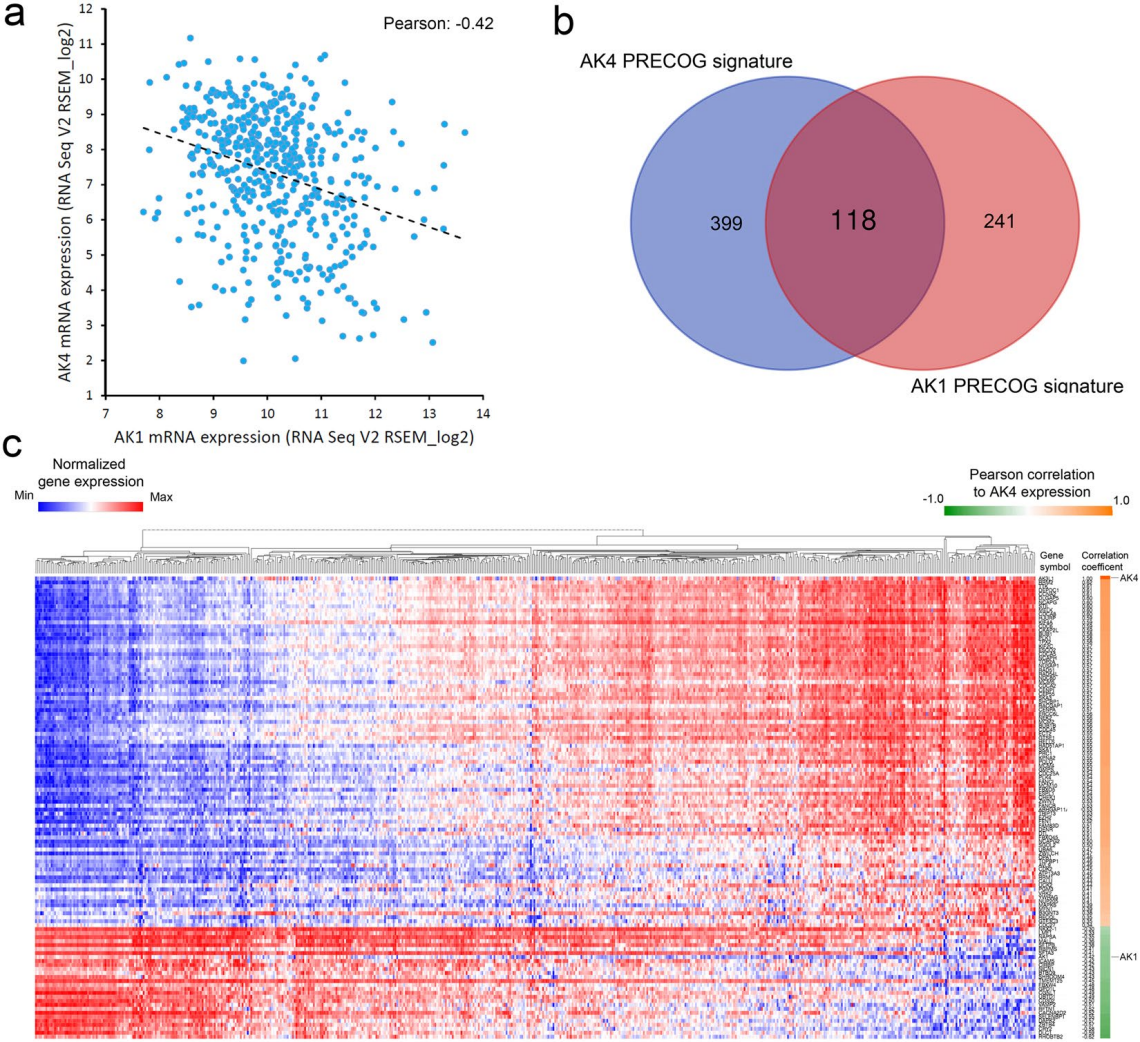
AK4-AK1 PRECOG gene signature predicts patient survival in lung adenocarcinomas. (**a**) Correlation between AK4 and AK1 mRNA expression in TCGA LUAD RNA Seq dataset. (**b**) Venn diagram analysis of AK4 PRECOG gene signature and AK1 PRECOG gene signature. (**c**) Hierarchical clustering analysis of consensus AK4-AK1 PRECOG gene signature.

Through analyzing RNA expression, we found AK4 gene expression negatively correlated with AK1 gene expression in TCGA LUAD RNA Seq dataset (Pearson correlation coefficient = −0.42; Fig. 3a). We next conduct Venn diagram analysis between AK4 PRECOG signature and AK1 PRECOG signature and identified a consensus 118-gene set (Fig. 3b). To illustrate global gene expression pattern, we performed cluster hierarchy analysis on 118 genes in TCGA LUAD RNA Seq dataset. Heatmap showed the ranked genes according to Pearson correlation to AK4 expression (Fig. 3c) and AK1 expression (Supplement Fig. S2).

**Figure 4:**
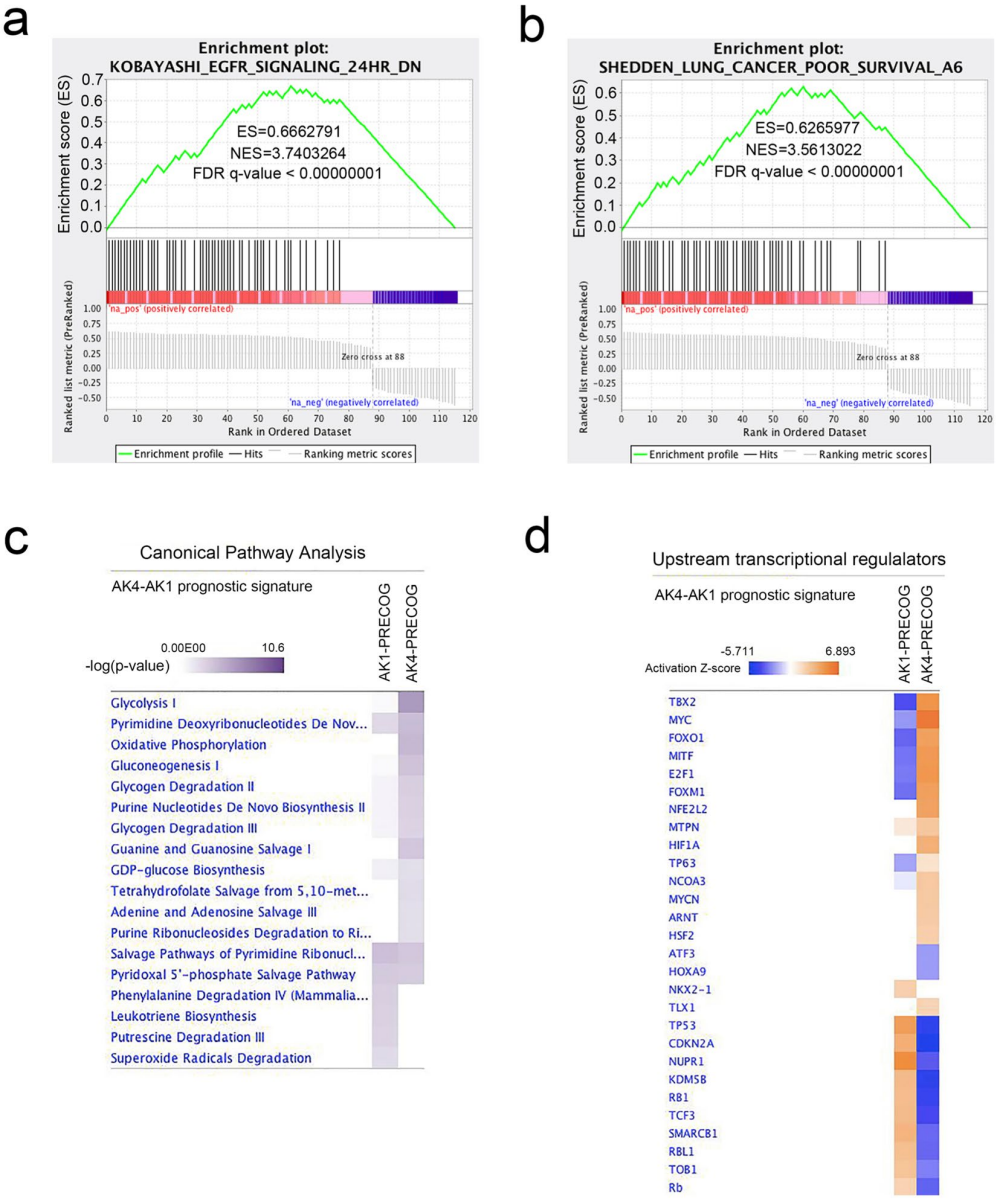
Gene set enrichment analysis (GSEA) and IPA pathway analysis of consensus AK4-AK1 PRECOG gene signature. GSEA plot of KOBAYASHI_EGFR_SIGNALING_24HR_DN gene set (**a**) and SHEDDEN_LUNG_CANCER_POOR_SURVIVAL_A6 gene set (**b**) in consensus AK4-AK1 PRECOG signature. (**c**) IPA Canonical pathway analysis of AK4-AK1 PRECOG gene signatures identifies altered metabolic pathways in lung adenocarcinoma patients. (**d**) IPA Upstream analysis of AK4-AK1 PRECOG gene signatures predicts activation or inhibition status of transcription regulators in lung adenocarcinomas. Transcription factors with activation Z-score ≥ 2 or ≤ −2 were predicted to be activated or inhibited respectively.

To gain more functional insight, we performed gene set enrichment analysis of AK4-AK1 PRECOG gene signature using C2: curated gene sets as background^33,34^. The result showed many upregulated genes in the signature were exclusively enriched in genes downregulated 24 hours after EGFR inhibitor treatment in Gefitinib-resistant lung cancer cells (Fig. 4a and Supplementary Table S3)^35^. Moreover, GSEA also confirmed the prognostic nature of AK4-AK1 gene signature as nearly 50% of the genes in the gene signature were enriched in lung cancer poor survival gene sets reported by Director’s Challenge Consortium for the Molecular Classification of Lung Adenocarcinoma (Fig. 4b and Supplementary Table S3)^29^. IPA Canonical pathway analysis revealed that vast majorities of AK4- and AK1-associated prognostic genes were involved in metabolic processes such as glucose metabolism, nucleotide/energy homeostasis and oxidative stress response (Fig. 4c and Supplementary Table S4). Furthermore, IPA upstream analysis predicted the activation or inhibition state of many important transcription factors that were previously reported to be involved in the pathogenesis of lung cancer such as FOXM1, NRF2, TP63, NKX2-1, and ATF3 (Fig. 4d and Supplementary Table S4)^18,36–39^. Taken together, these data showed AK4-AK1 consensus gene signature has significant impact on the survival of lung adenocarcinoma patients and may represent an important signaling axis that affect the pathogenesis of lung adenocarcinomas and the response to EGFR inhibition.

### Reduced dependency of EGFR signaling by decreasing AK4 in lung adenocarcinoma cells

**Figure 5:**
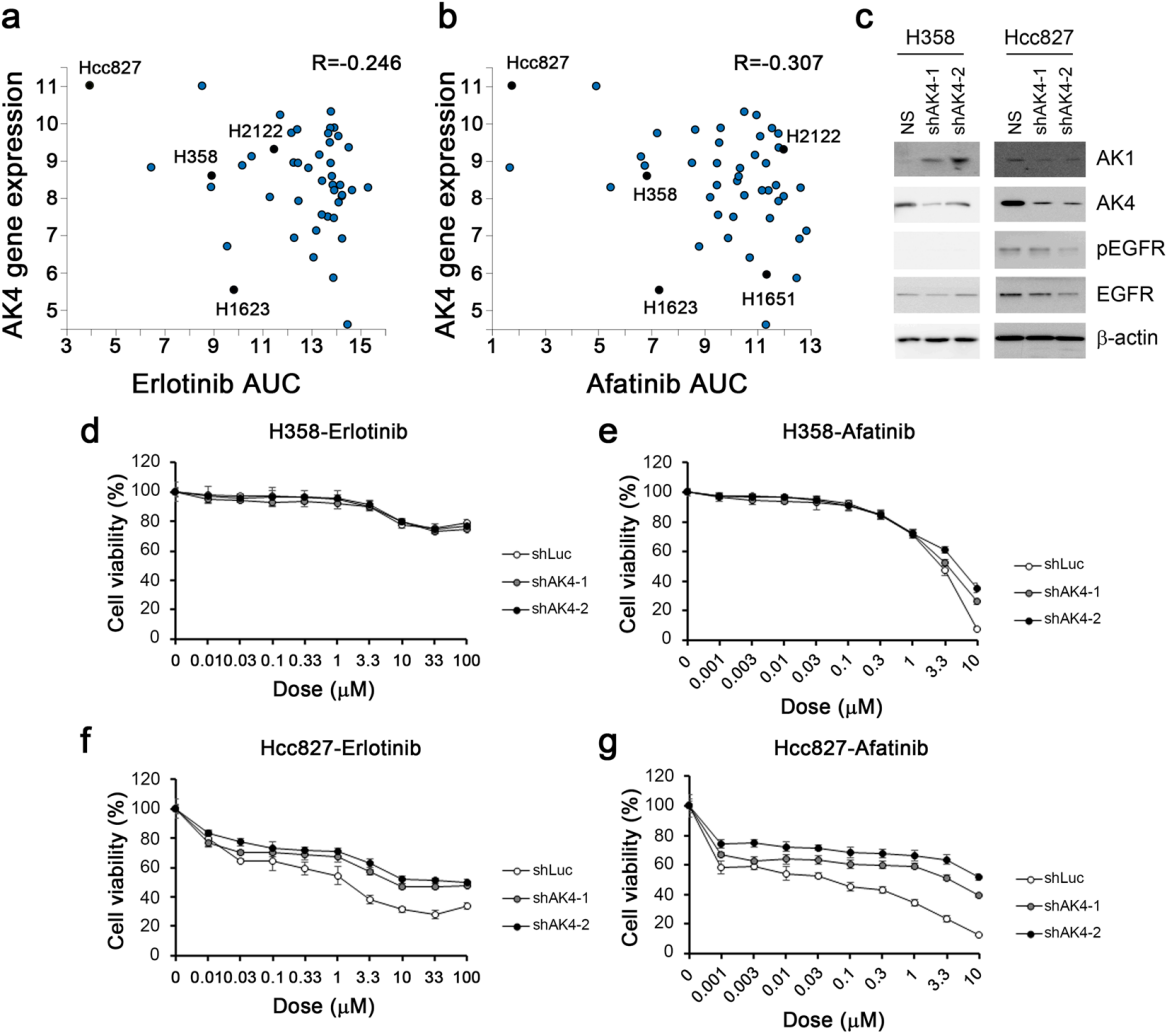
AK4 signaling helped the response of EGFR inhibitors. (**a**) Correlation plot of AK4 expression level and AUC of Erlotinib in COSMIC lung adenocarcinoma cell lines. Specific cell lines were presented in black. (**b**) Correlation plot of AK4 expression level and AUC of Erlotinib in COSMIC lung adenocarcinoma cell lines. Specific cell lines were presented in black. (**c**) The expression level of AK1, AK4, pEGFR, and EGFR in H358 and Hcc827 cells after shAK4 treatment. Beta-actin was used as loading control. (**d**) The cell viability assay of Erlotinib in H358-shLuc, H358-shAK4-1 and H358-shAK4-2 cells. (**e**) The cell viability assay of Afatinib in H358-shLuc, H358-shAK4-1 and H358-shAK4-2 cells. (**f**) The cell viability assay of Erlotinib in Hcc827-shLuc, Hcc827-shAK4-1 and Hcc827-shAK4-2 cells. (e) The cell viability assay of Afatinib in Hcc827-shLuc, Hcc827-shAK4-1 and Hcc827-shAK4-2 cells.

Active glycolysis increased the survival through EGFR signal and reducing receptor degradation in EGFR mutant lung cancer^40^. AK4 overexpression enhanced the glycolysis (Fig. 4c). The linkage between AK4 and EGFR has never mentioned before. AK4 overexpression has correlated to poor prognosis and metastasis in lung cancer thus the inhibition of AK4 might provide a potential therapeutic target. We initially assessed the relationship between AK4 and therapeutic drugs on cancer therapeutics response portal (CTRP v2, and Supplement Fig. S3a). The data shown the IC50 of EGFR inhibitors, erlotinib and afatinib, have negative correlation with the expression of AK4 (Fig. 5a,b). The cytotoxicity results of Hcc827 and H358, which have high AK4 expression, were sensitive to EGFR inhibitors in our cell collection (Supplement Fig. S3b). H2122 cell has high AK4 RNA expression but low amount in protein level and has low response to EGFR inhibitors (Fig. 5a). In EGFR wildtype H358 after AK4 downregulation, AK1 expression increased and no obvious EGFR changes. In EGFR mutation Hcc827 cell, AK1 and EGFR expressions downregulated (Fig. 5c). In cell viability assay, the data showed that the responses to EGFR inhibitors, Erlotinib and Afatinib, were decreased after AK4 protein reducing (Fig. 5d–g). The drug resistances were increased with the trend of the reducing level of EGFR, especially in EGFR mutation cell line, Hcc827. Therefore, AK4 signaling might involve in stabilizing the EGFR protein and its signaling in cells. However, the exact mechanisms and drug targets that associated with AK4-AK1 status remains to be validated.

## Discussion

In this study, we have identified a consensus gene set derived from AK4- and AK1-associated gene expression signature in lung adenocarcinoma. This unique gene set not only dictated patient outcomes but also related to the sensitivity to EGFR inhibition. Moreover, upstream regulator and pathway analyses further revealed putative transcription factor networks and metabolic pathways that may impact the survival of lung adenocarcinoma patients.

The expression of AKs forms an integrated signaling network that connects energetic demand/supply and metabolic processes. Here we identify a unique AK4-AK1 axis that dictates the outcomes of lung adenocarcinoma patients. In our analyses, we found overexpression of AK4 and downregulation of AK1 are associated with poor prognosis in lung adenocarcinoma. Spatial repositioning of AK1 has been shown to provide local ATP supply to fuel energy-consuming cellular processes such as actomyosin machinery^41^. On the other hand, AK4 was reported to interact with ADP/ATP translocator and voltage-dependent anion channel (VDAC) at mitochondrial matrix, and these interactions are required for the regulation of mitochondria membrane permeability and the exchange of ADP/ATP between mitochondrial matrix and cytosol^17^. Therefore we hypothesized that lung cancer cells with AK4-high/AK1-low represent a status of restricted energy demand as pathway and upstream regulator analyses revealed glycolysis and HIF-1α were the predominant metabolic pathway/transcription factor of the gene signatures (Fig. 4c,d). In actively proliferating cancer cells, aerobic glycolysis is preferable despite a less efficient metabolism for ATP production. However, cells undergoing aerobic glycolysis often use other metabolic pathways to maintain high ratios of ATP/ADP and NADH/NAD+^42^. AK4 was identified as key regulator of cellular ATP levels in a mitochondrial RNAi screening as AK4 knockdown increases ATP levels by more than 25 percent^43^. However, cells maintain ATP, ADP, and AMP levels at ratios of 100:10:1, the conversion of ADP plus AMP to restore ATP can only increase ATP levels around 10 percent. Therefore activation of adenosine biosynthesis may effectively maintain high ratios of ATP/ADP when oxidative phosphorylation is compromised^44,45^. These studies are consistent with our pathway analyses that enzymes in nucleotide *de novo* biosynthesis were significantly enriched in AK4-AK1 PRECOG gene signatures (Fig. 4c).

As the major type of lung cancer, NSCLC is further characterized by a high degree of pathological heterogeneity including adenocarcinoma (ADC, ~48%), squamous cell carcinoma (SCC, ~28%), and large cell carcinoma (LCC, ~24%). Generally, ADCs arise from alveolar epithelial cells and occur in distal airways, whereas SCCs arise from basal cell and occur in proximal airways^46,47^. Typical ADCs have glandular histology and express thyroid transcription factor 1 (TTF-1, gene symbol: *NKX2-1*) and keratin 7 as biomarkers. On the other hand, SCCs express basal cell markers p63 (gene symbol: *TP63*) and keratin 5/14. However, the definitive markers that define the pathology of ADC and SCC remain to be determined. For example, approximately 15–20% of ADCs do not express TTF-1 and these patients are often associated poor outcome due to the lack of druggable mutations or molecular targets^48,49^. Moreover, the mixed ADC and SCC pathology with identical mutations has been frequently found in human lung tumors known as adenosquamous cell carcinoma^50–52^. This lineage plasticity was reported in mice with Lkb1 deficiency^53^. Moreover, LKB1 inactivation in Kras-driven NSCLC promotes ADC to transdifferentiate into p63-positve SCC through metabolic alterations including increased oxidative stress and develops resistant to therapy^54^. By analyzing gene expression signatures of AK family in TCGA lung adenocarcinomas, we surprisingly found that target genes of “SCC” marker p63 (*TP63*) were increasingly upregulated upon the over-expression of AK4 and the under-expression of AK1. By contrast, the activation of TTF-1 (*NKX2-1*) was positively associated with AK1 expression (Fig. 4d). Notably, we also found AK4- and AK1-associated signatures were mainly involved in metabolic processes including nucleotide/energy homeostasis, oxidative stress response, and glucose metabolism (Fig. 4c). These data suggest metabolic reprogramming that associated with AK4- and AK1-mediated bioenergetics changes may be critical for the pathogenesis of ADC-to-SCC lineage transdifferentiation.

Accumulating evidence has shown the control of metabolic reprograming is tightly linked to oncogene/tumor suppressor signaling^55^. Particularly, a recent study reported EGFR mutation enhances glycolysis to maintain cell survival by inhibiting EGFR autophagy-mediated degradation in lung adenocarcinoma cells^40^. EGFR-TKI treatment decreases glycolysis metabolism in lung adenocarcinoma harbor EGFR mutations^56^. Accordingly, we also observed a vast majority of upregulated genes in consensus gene set of AK4- and AK1- gene signatures are enriched in the downregulated gene set upon EGFR inhibition (Fig. 4a). Moreover, our data showed the amount of EGFR protein decreased with reducing AK4 expression and make cells more EGFR signaling independent and reduced the sensitivity to the EGFR inhibitors (Fig. 5). However, it requires further study to determine whether modulation of AK4 and/or AK1 may overcome T790M-mediated resistance, and through what mechanisms adenylate kinases isoform network may modulate EGFR signaling turn over in lung cancer.

## Supplementary information

Supplementary figures

Table S1

Table S2

Table S3

Table S4

raw data of blots
