# Supplementary material for: A co-expressed gene status of adenylate kinase 1/4 reveals prognostic gene signature associated with prognosis and sensitivity to EGFR targeted therapy in lung adenocarcinoma
Source: Sci Rep. 2019 Aug 23;9:12329. doi: 10.1038/s41598-019-48243-9 (PMC6707279; doi:10.1038/s41598-019-48243-9)
Supplement: Supplementary file 1 — Supplementary figures [file 41598_2019_48243_MOESM1_ESM.pdf]

Supplementary information

A co-expressed gene status of adenylate kinase 1/4 reveals prognostic gene signature associated with prognosis and sensitivity to EGFR targeted therapy in lung adenocarcinoma

Yi-Hua Jan[1*,] Tsung-Ching Lai[1*], Chih-Jen Yang[2], Ming-Shyan Huang[4#], and Michael Hsiao[1, 3#]

[1]Genomics Research Center, Academia Sinica, Taipei, Taiwan, [2] Department of Internal Medicine, Kaohsiung Medical University Hospital, School of Medicine, Kaohsiung Medical University, Kaohsiung, Taiwan, [3]Department of Biochemistry, College of Medicine, Kaohsiung Medical University, Kaohsiung, Taiwan. [4]School of Medicine, I-Shou University, Kaohsiung, Taiwan.

*The first two authors contribute equally to this works.

[#]The last two authors are senior authors and contribute equally to this works.

Correspondence to: Dr. Michael Hsiao, Genomics Research Center, Academic Sinica, 128 Academia Road, Section 2, Taipei 115, Taiwan. Phone: 886-2-2787-1243; Fax: 886-2-2789-9931; E-mail: mhsiao@gate.sinica.edu.tw

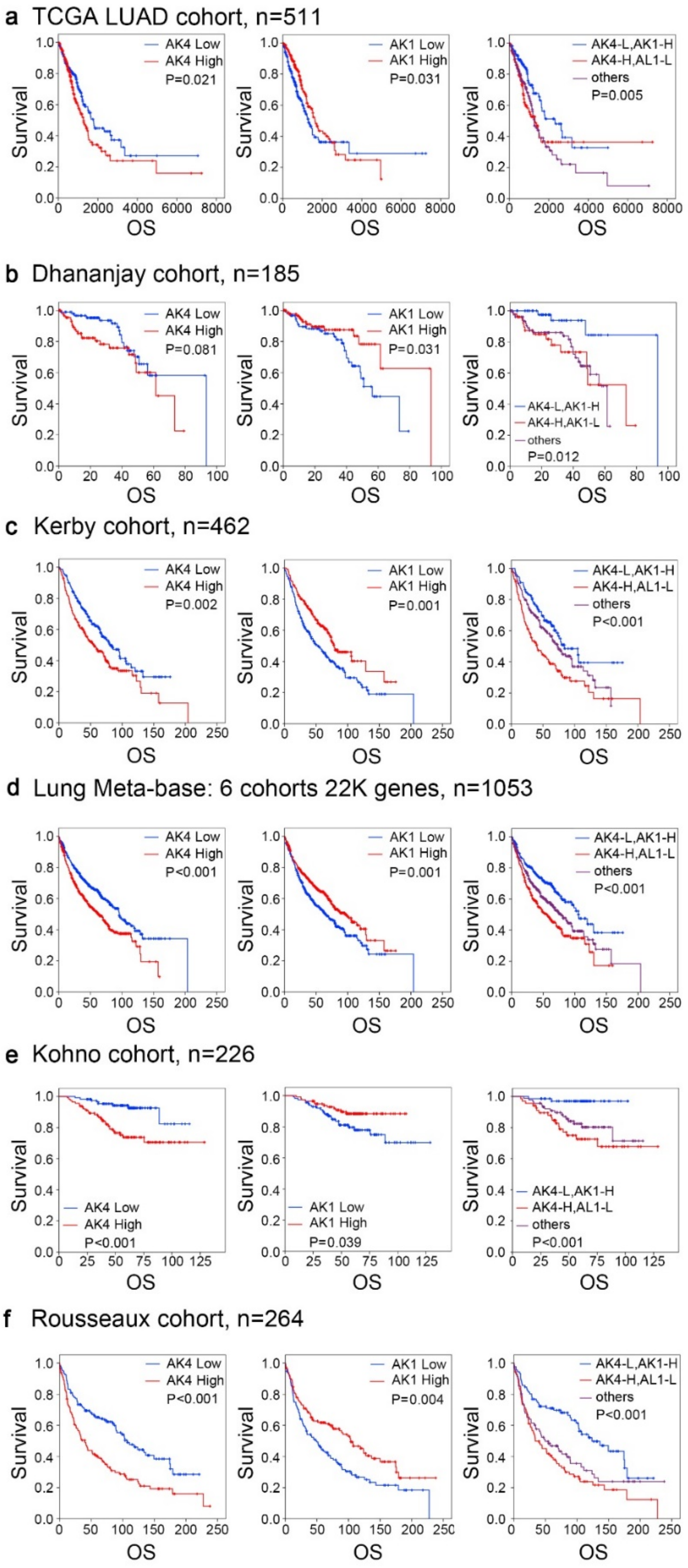

Figure S1. Kaplan-Meier survival analyses of AK4 only (left panel), AK1 only (center panel), and AK4-AK1 combination (right panel) in variant cohorts. a. TCGA lung adenocarcinoma cohort, b. Dhananjay cohort, c. Kerby cohort, d. Lung meta-base cohorts, e. Kohno cohort, and Rousseaux cohort.

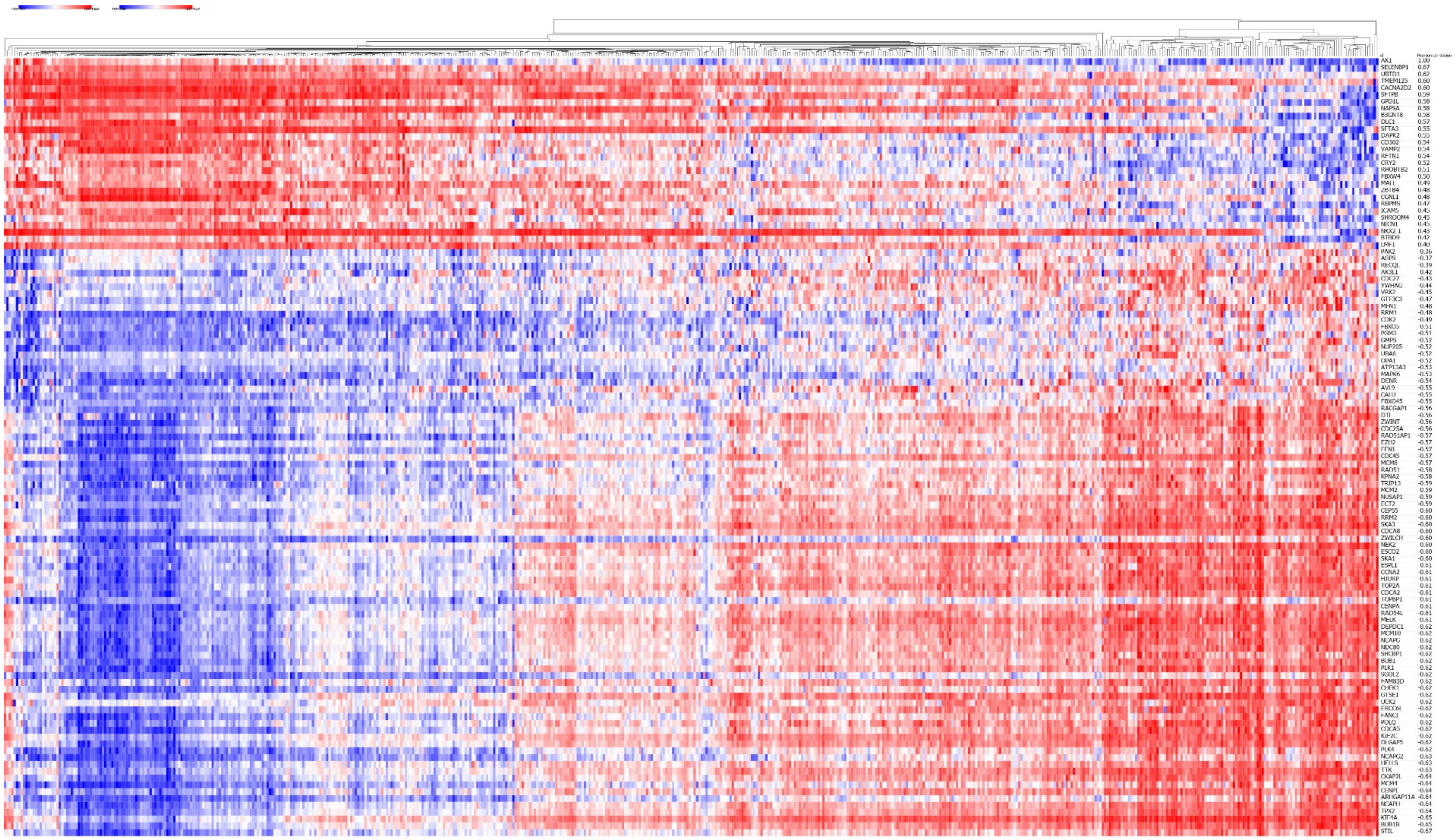

Figure S2. Hierarchical clustering analysis of consensus AK4-AK1 PRECOG gene signature. Ranked by AK1 expression.

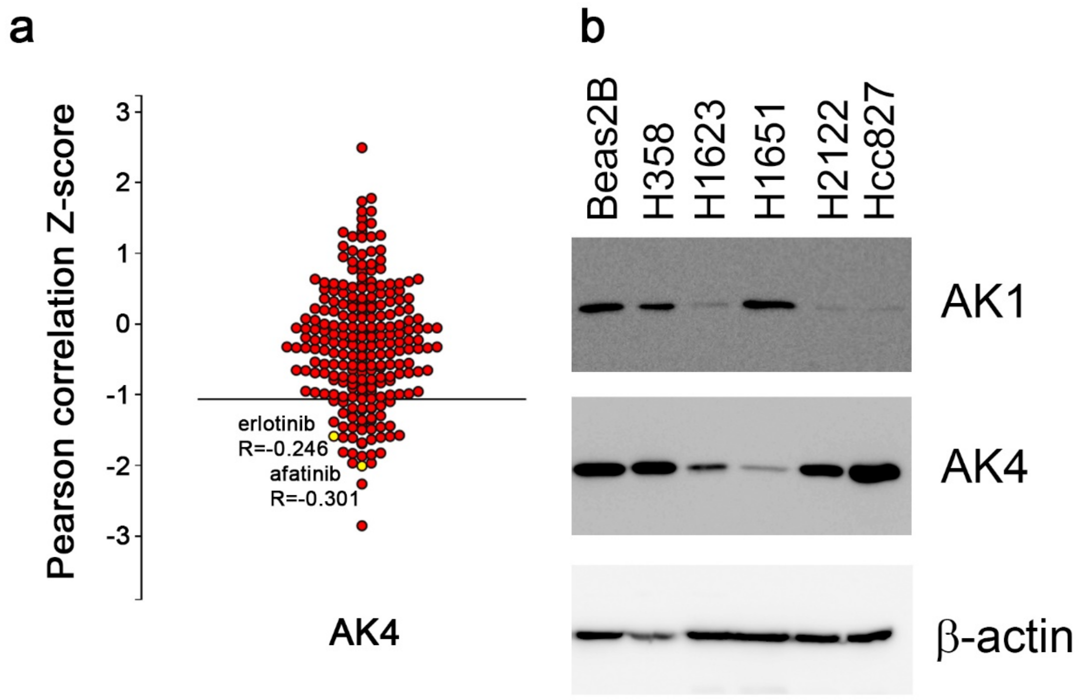

Figure S3. a. Distribution of pearson correlation Z-scroe between compound response and AK4 expression among lung adenocarcinoma cell lines from CTRP. b. AK1 and AK4 expression among lung cell lines by western blotting. Bease-2B is a normal bronchus epithelial cell line. Others are lung adenocarcinoma cell lines. Beta-actin is served as a loading control.
