# Supplementary material for: A co-expressed gene status of adenylate kinase 1/4 reveals prognostic gene signature associated with prognosis and sensitivity to EGFR targeted therapy in lung adenocarcinoma
Source: Sci Rep. 2019 Aug 23;9:12329. doi: 10.1038/s41598-019-48243-9 (PMC6707279; doi:10.1038/s41598-019-48243-9)

Supplementary information

A co-expressed gene status of adenylate kinase 1/4 reveals prognostic gene signature associated with prognosis and sensitivity to EGFR targeted therapy in lung adenocarcinoma

Yi-Hua Jan[1*,] Tsung-Ching Lai[1*], Chih-Jen Yang[2], Ming-Shyan Huang[4#], and Michael Hsiao[1, 3#]

[1]Genomics Research Center, Academia Sinica, Taipei, Taiwan, [2] Department of Internal Medicine, Kaohsiung Medical University Hospital, School of Medicine, Kaohsiung Medical University, Kaohsiung, Taiwan, [3]Department of Biochemistry, College of Medicine, Kaohsiung Medical University, Kaohsiung, Taiwan. [4]School of Medicine, I-Shou University, Kaohsiung, Taiwan.

*The first two authors contribute equally to this works.

[#]The last two authors are senior authors and contribute equally to this works.

Correspondence to: Dr. Michael Hsiao, Genomics Research Center, Academic Sinica, 128 Academia Road, Section 2, Taipei 115, Taiwan. Phone: 886-2-2787-1243; Fax: 886-2-2789-9931; E-mail: mhsiao@gate.sinica.edu.tw

Figure S3.

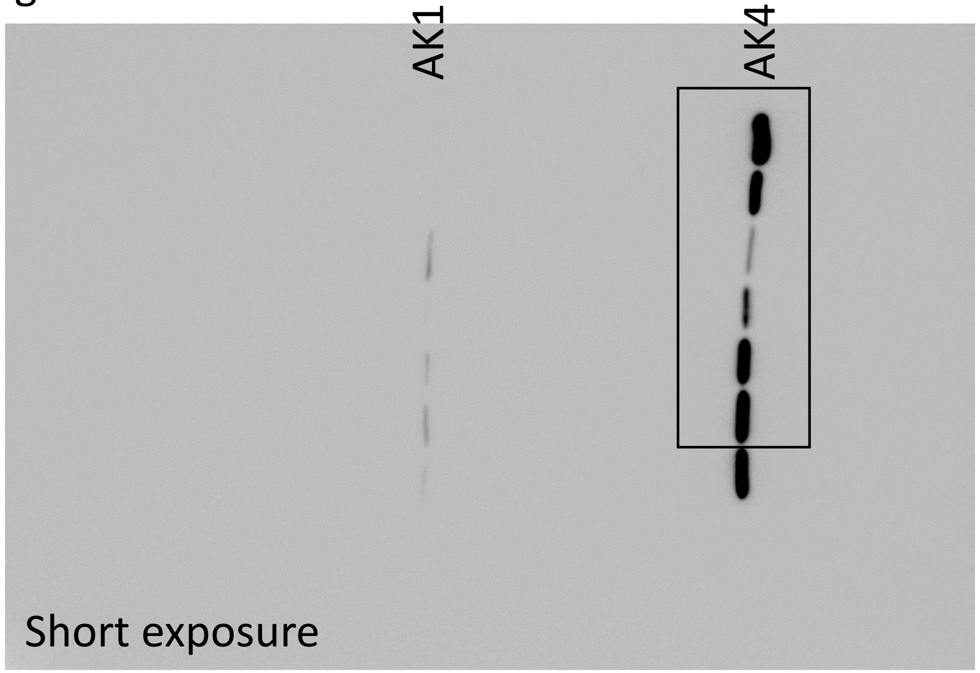
AK1
AK4
Short exposure

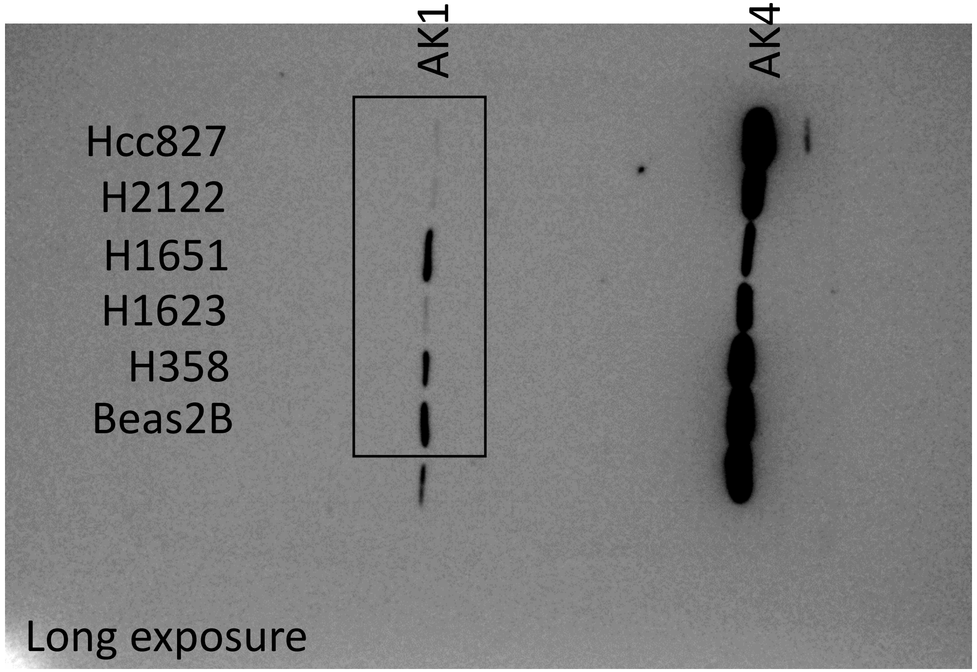
AK1
AK4
Hcc827
H2122
H1651
H1623
H358
Beas2B
Long exposure

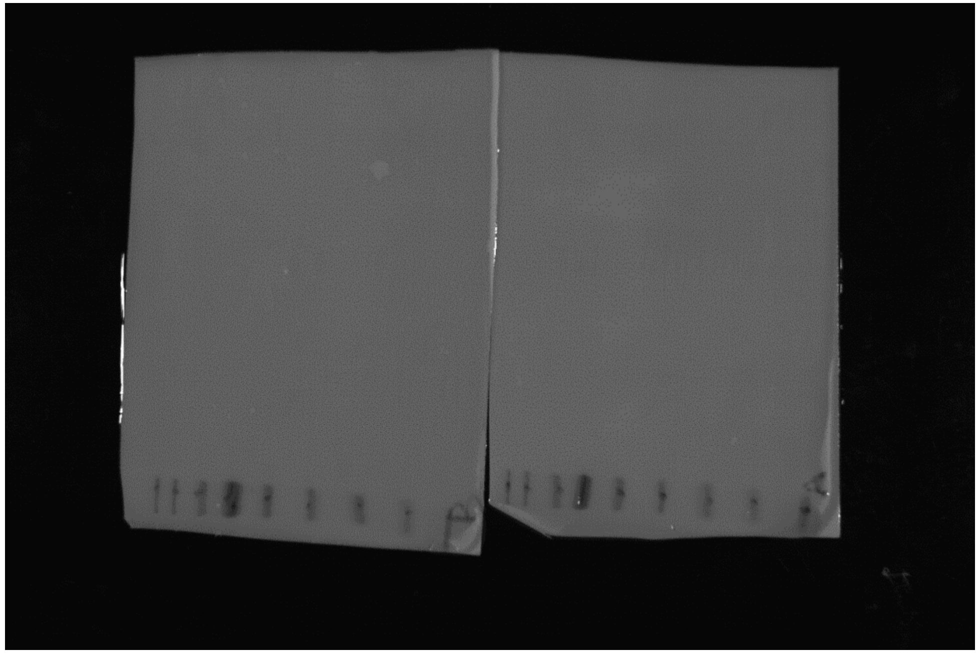

Figure S3.

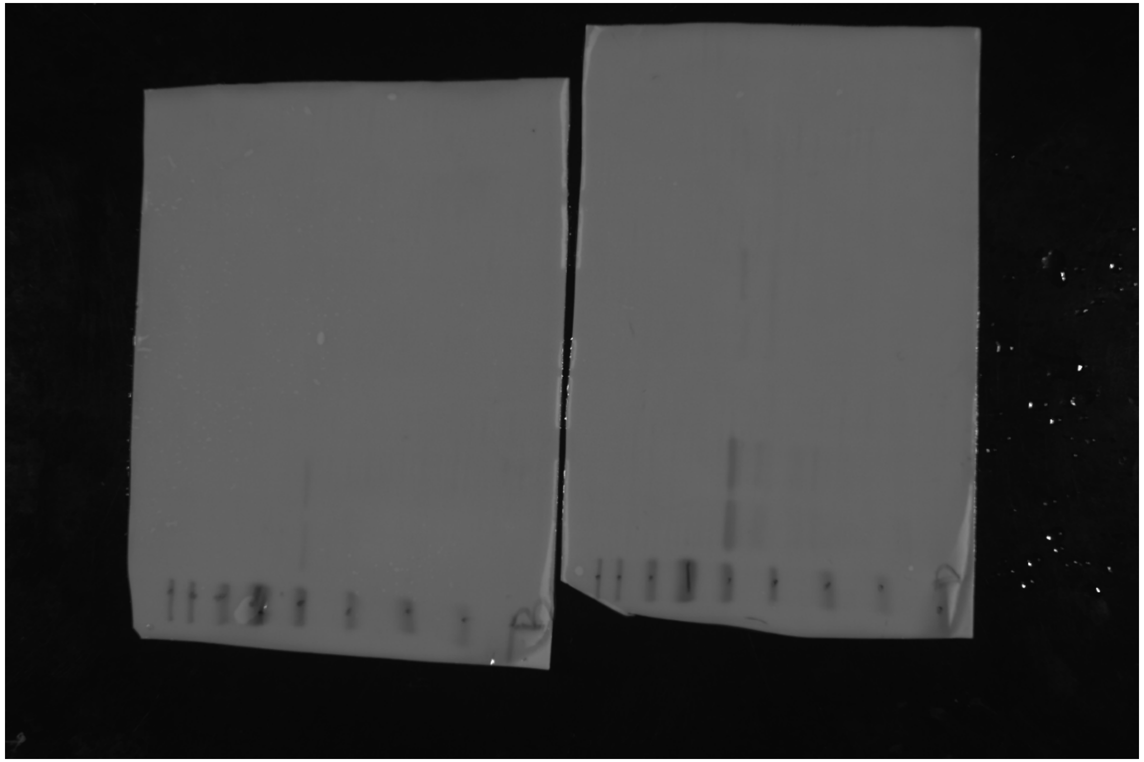

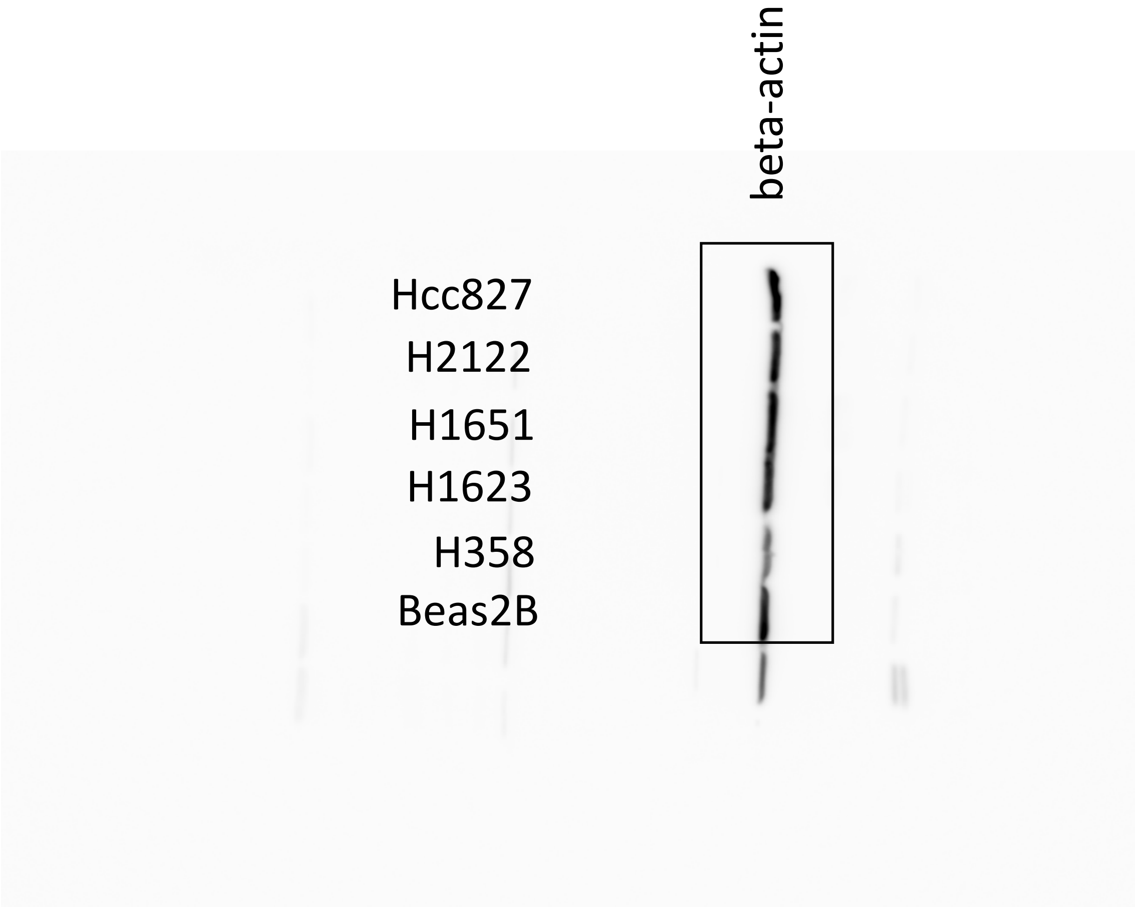

Fig 5.

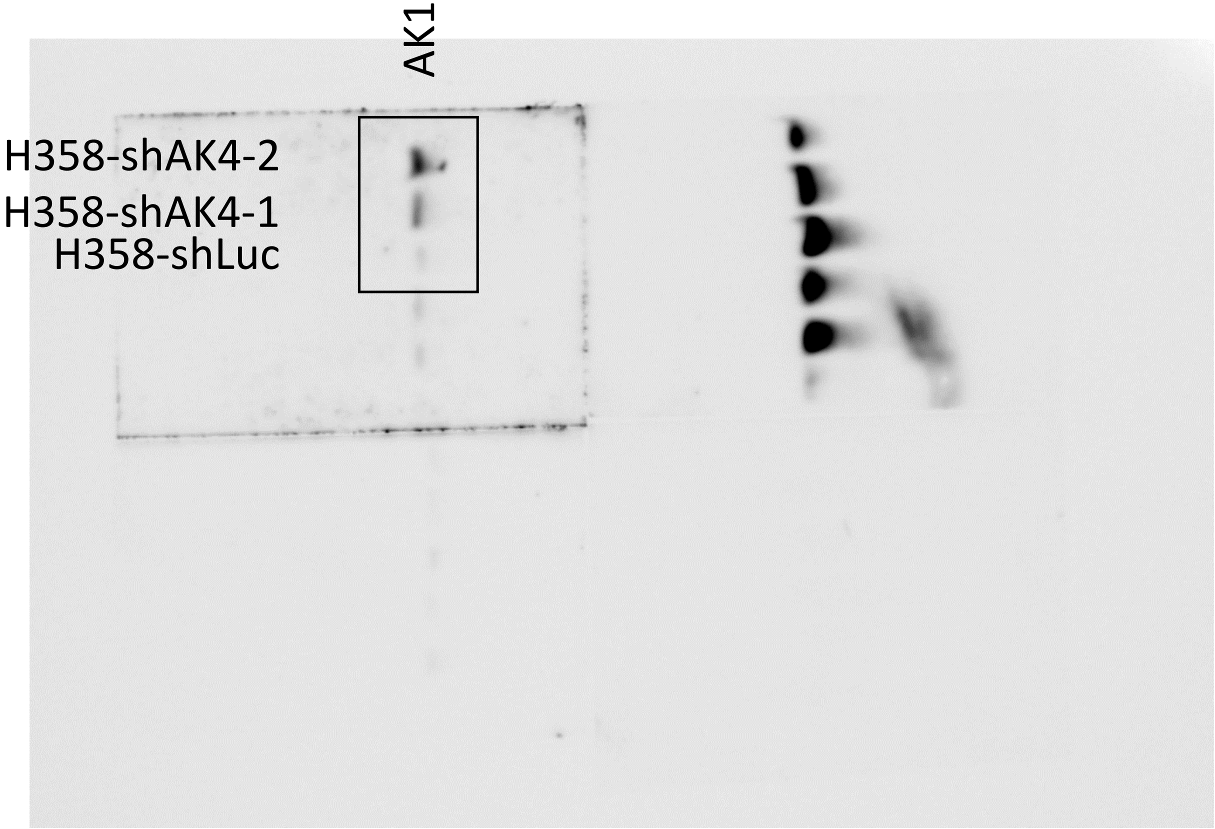

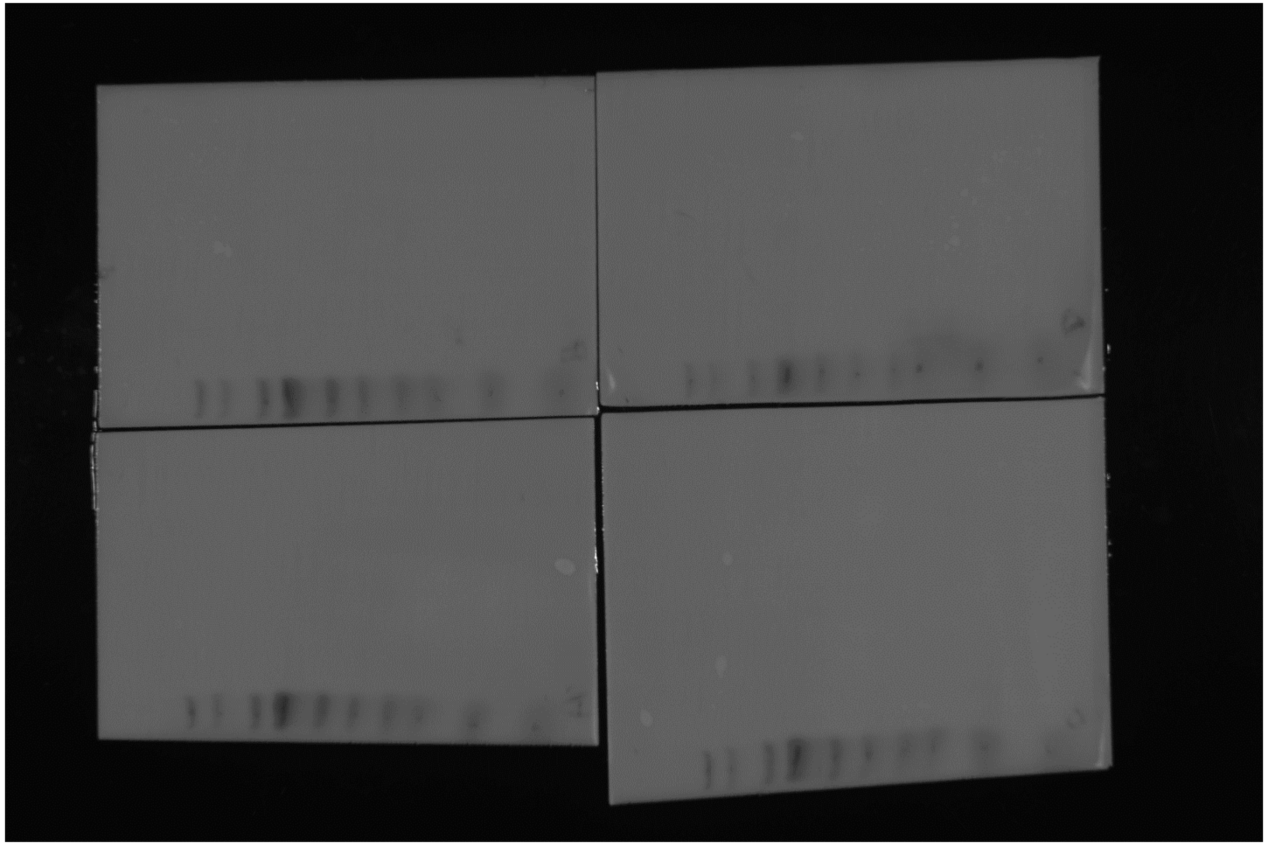

Fig 5.

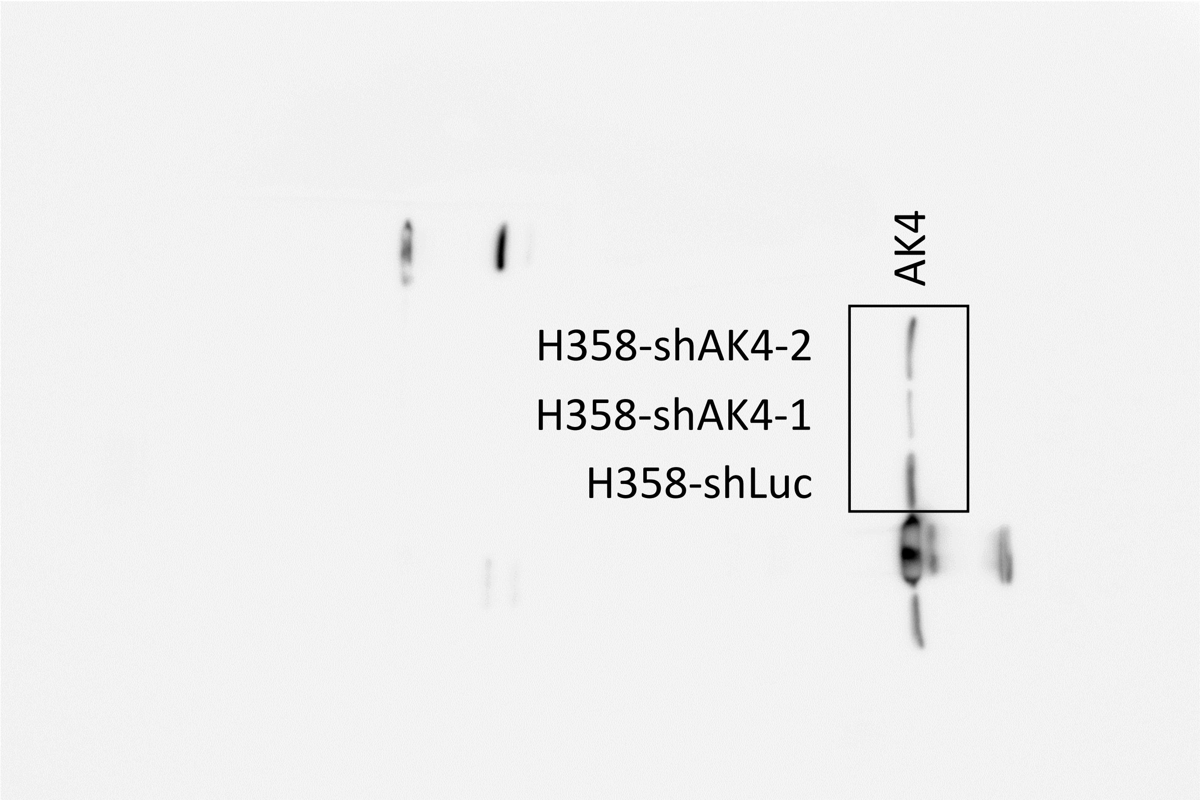

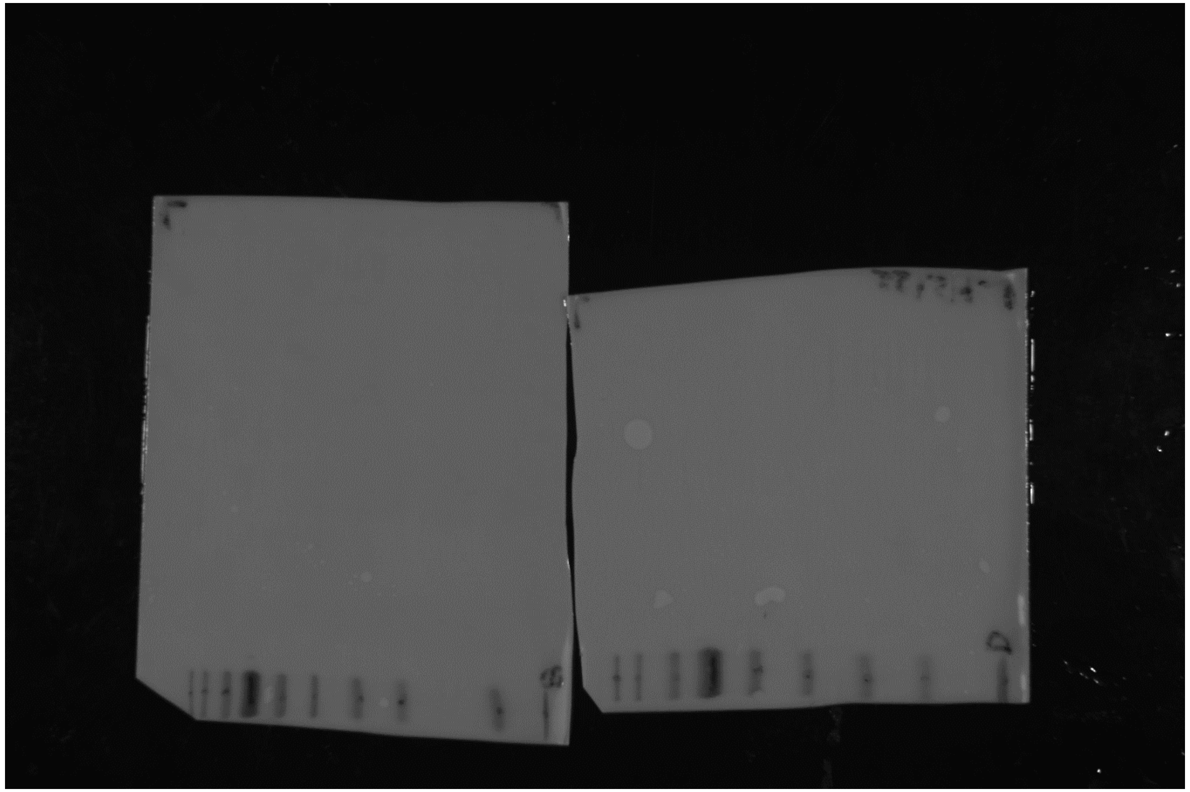

Fig 5.

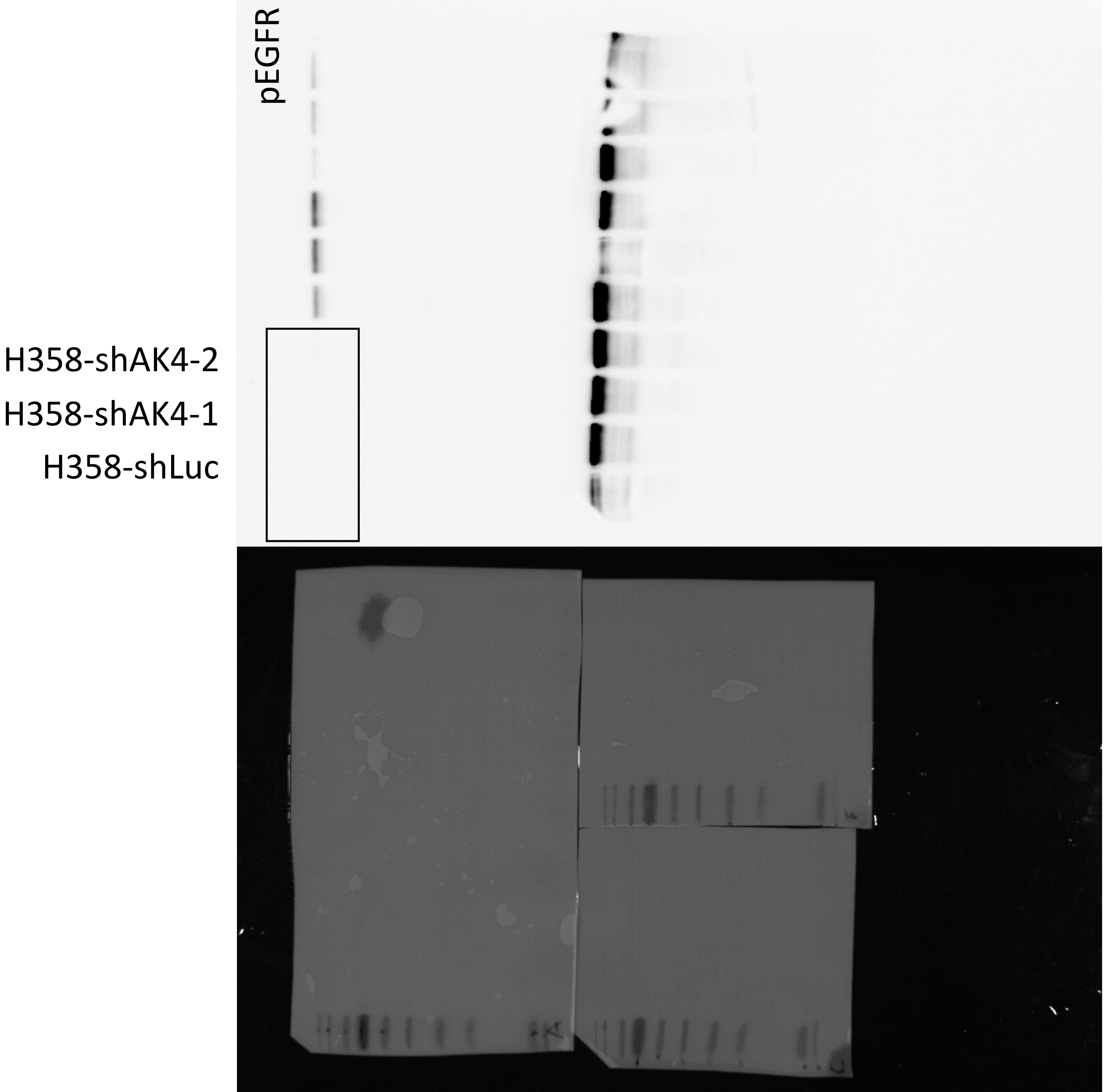

Fig 5.

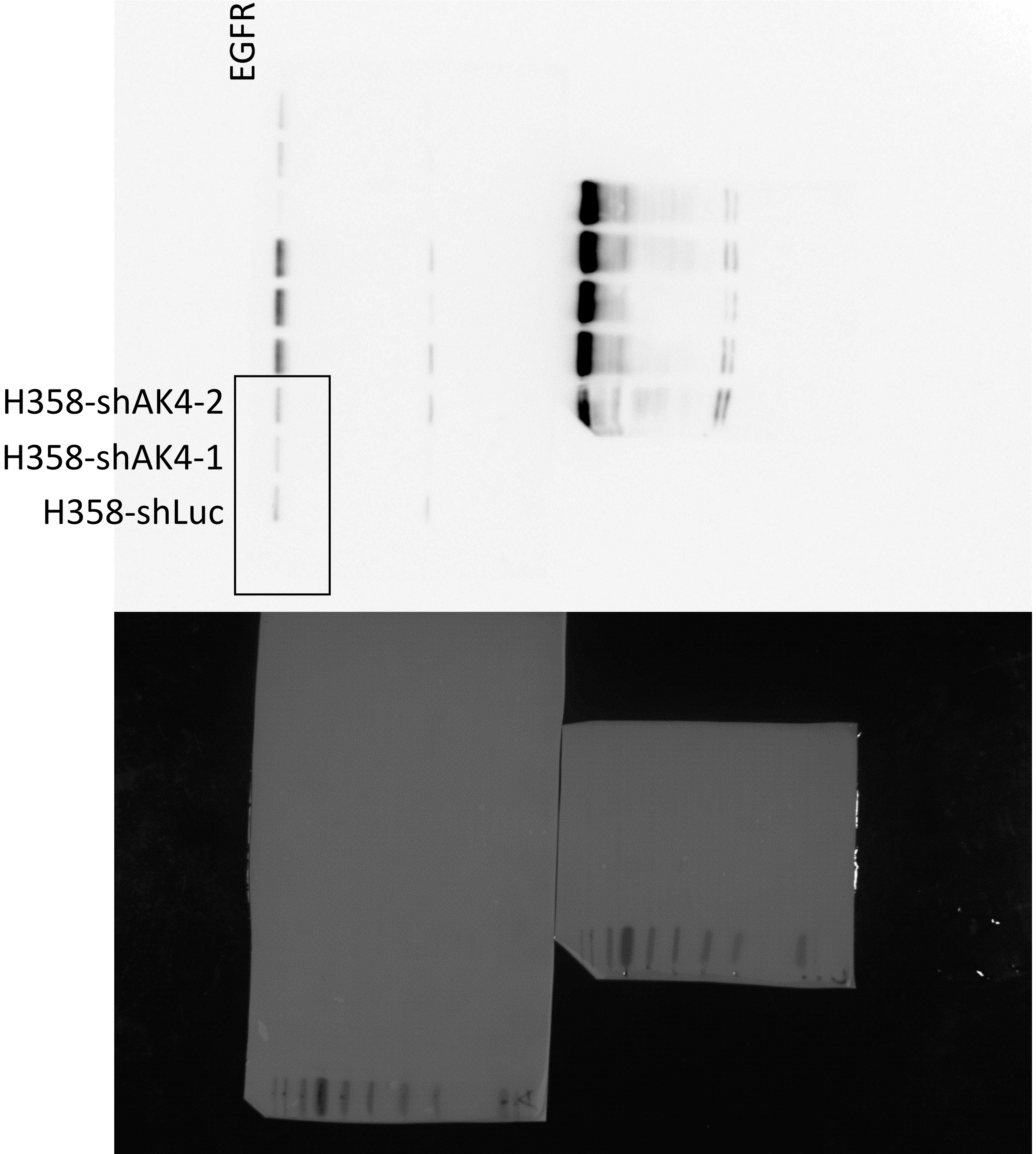

Fig 5.

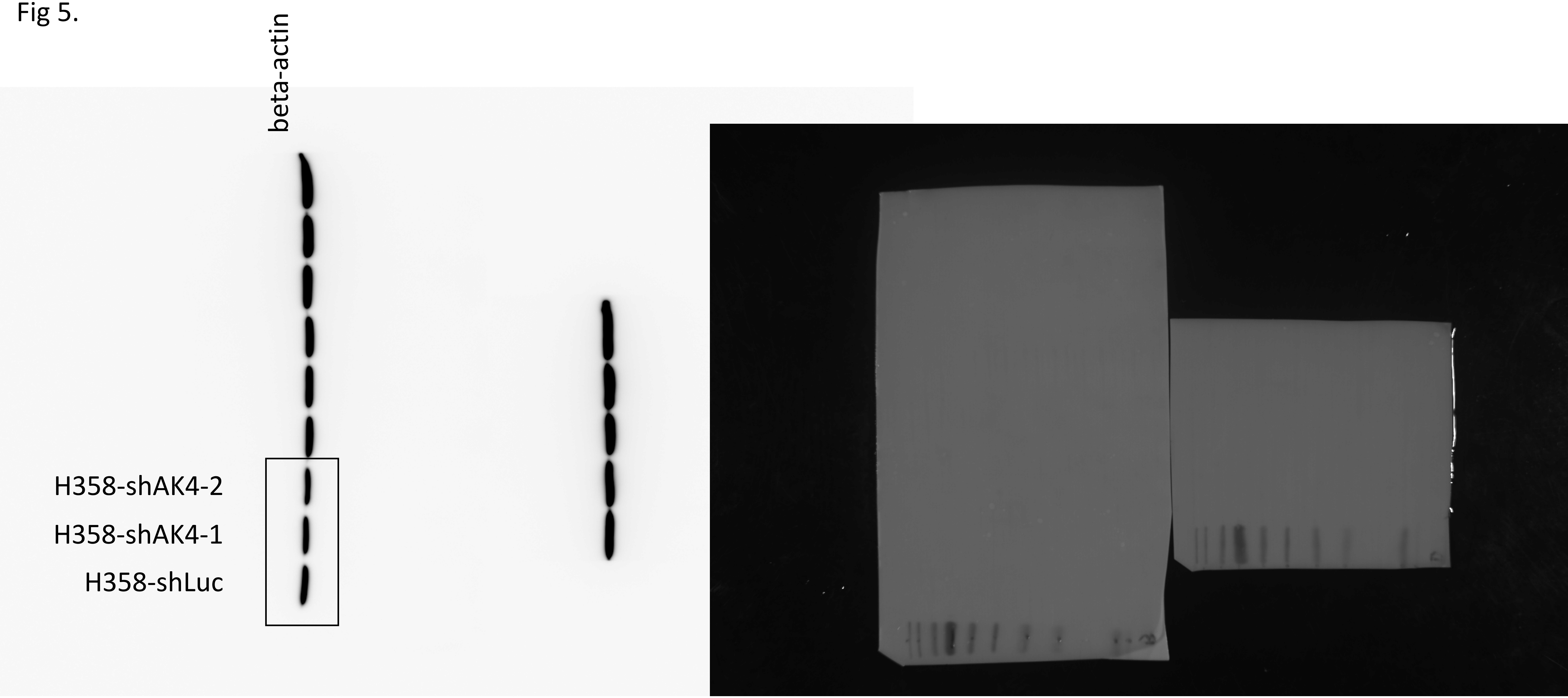

Fig 5.

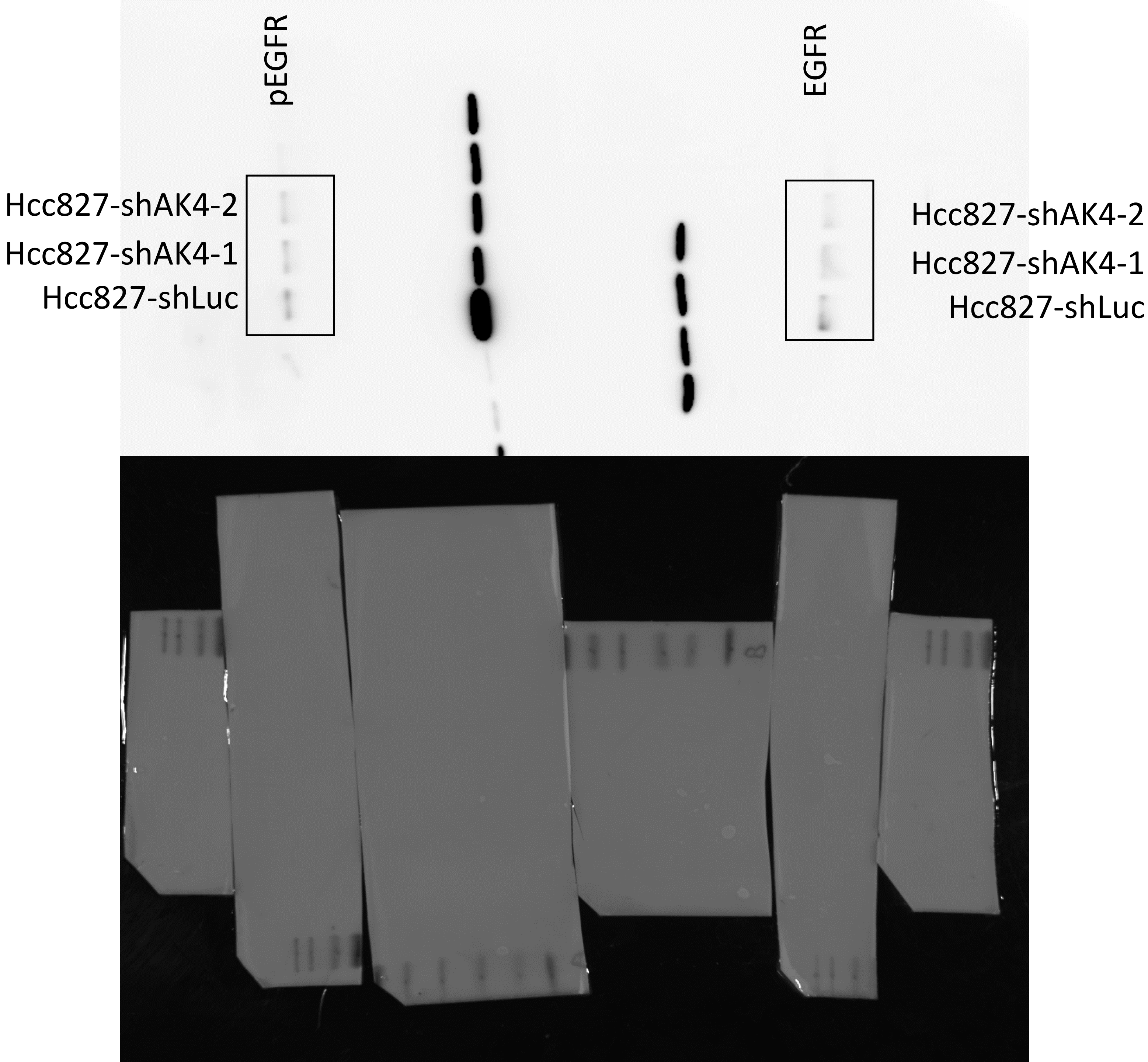
pEGFR
EGFR
Hcc827-shAK4-2
Hcc827-shAK4-1
Hcc827-shLuc
Hcc827-shAK4-2
Hcc827-shAK4-1
Hcc827-shLuc

Fig 5.

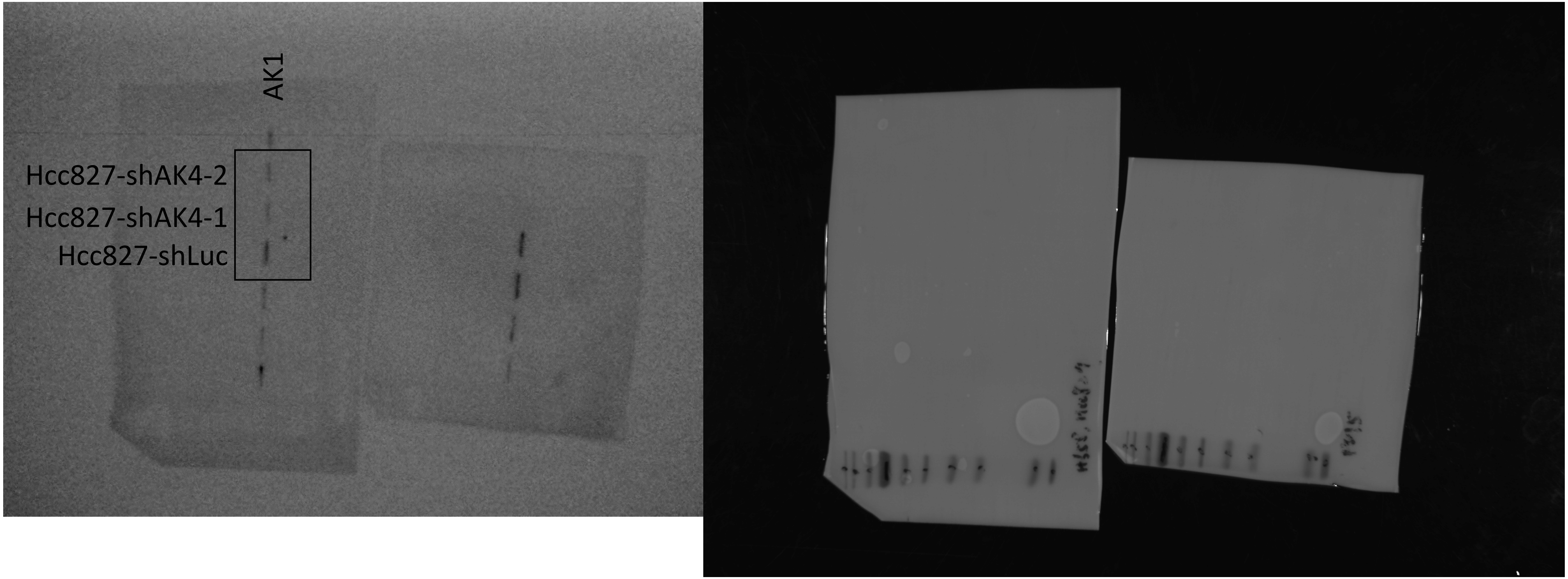

Fig 5.

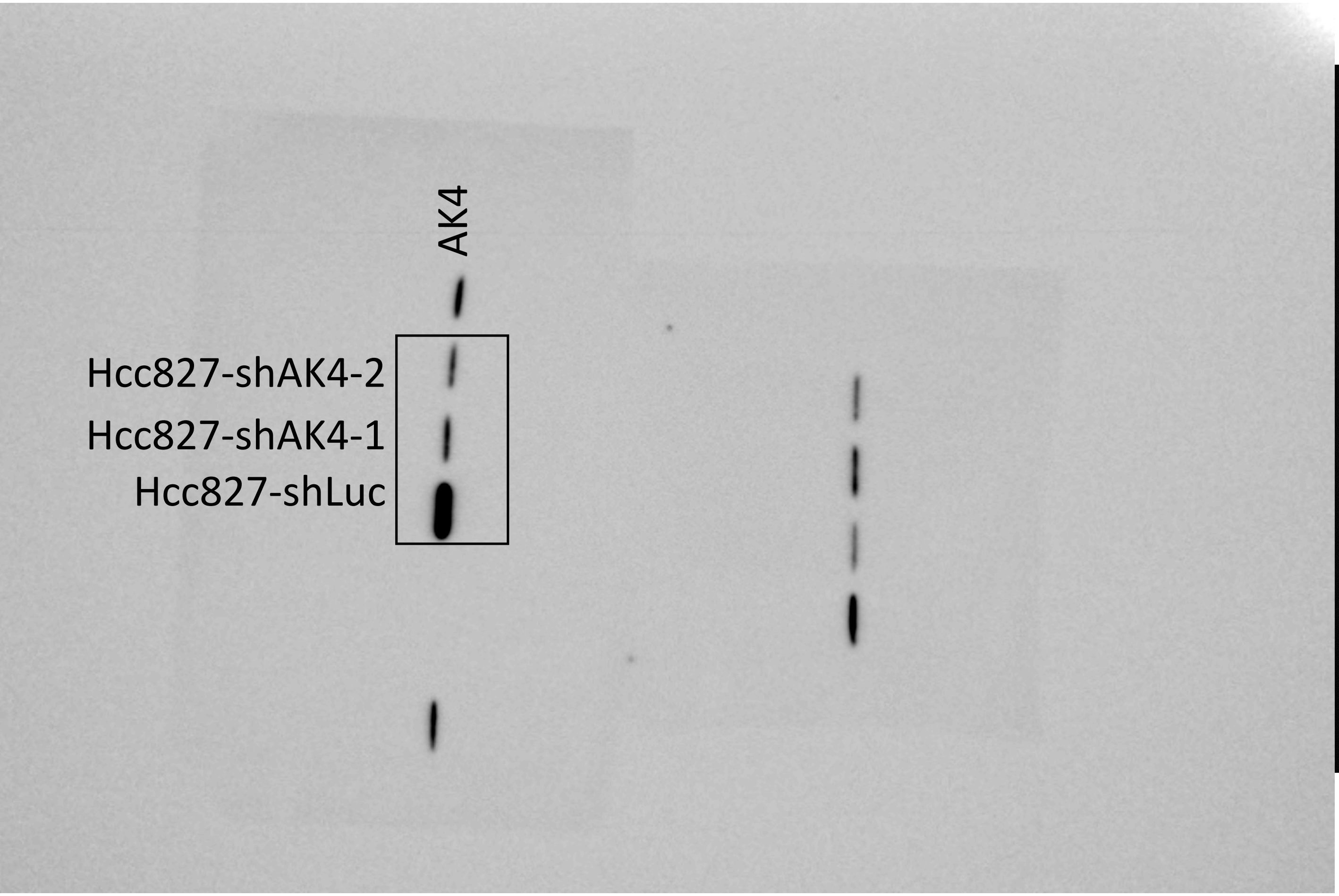

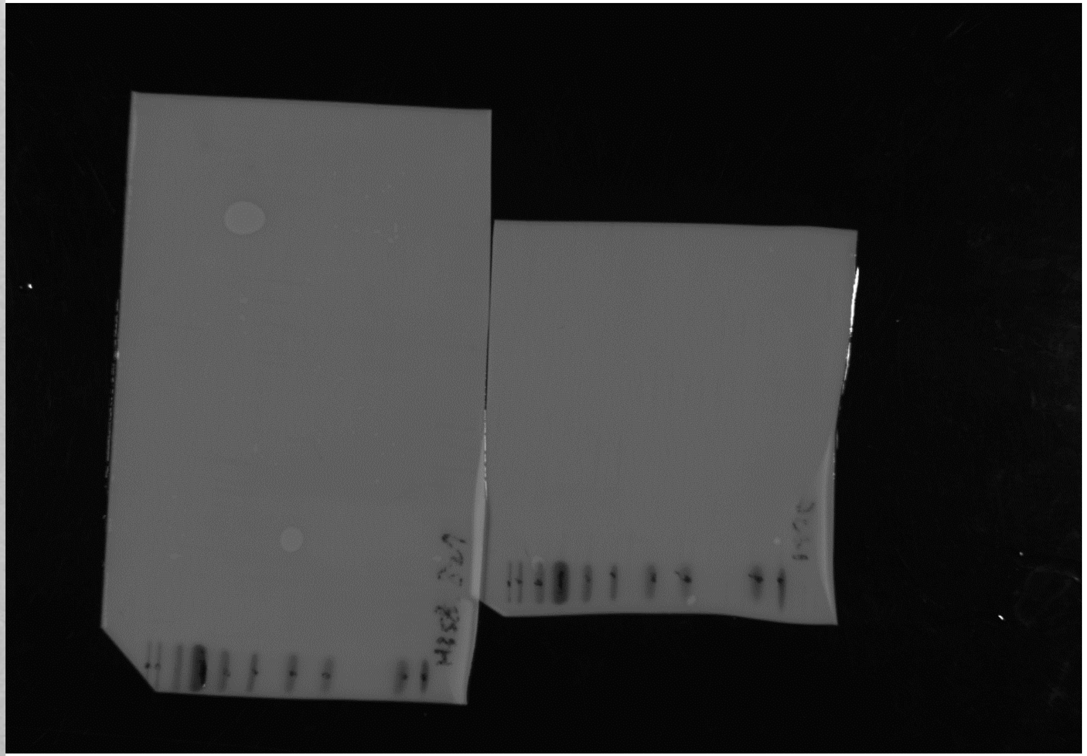

Fig 5.

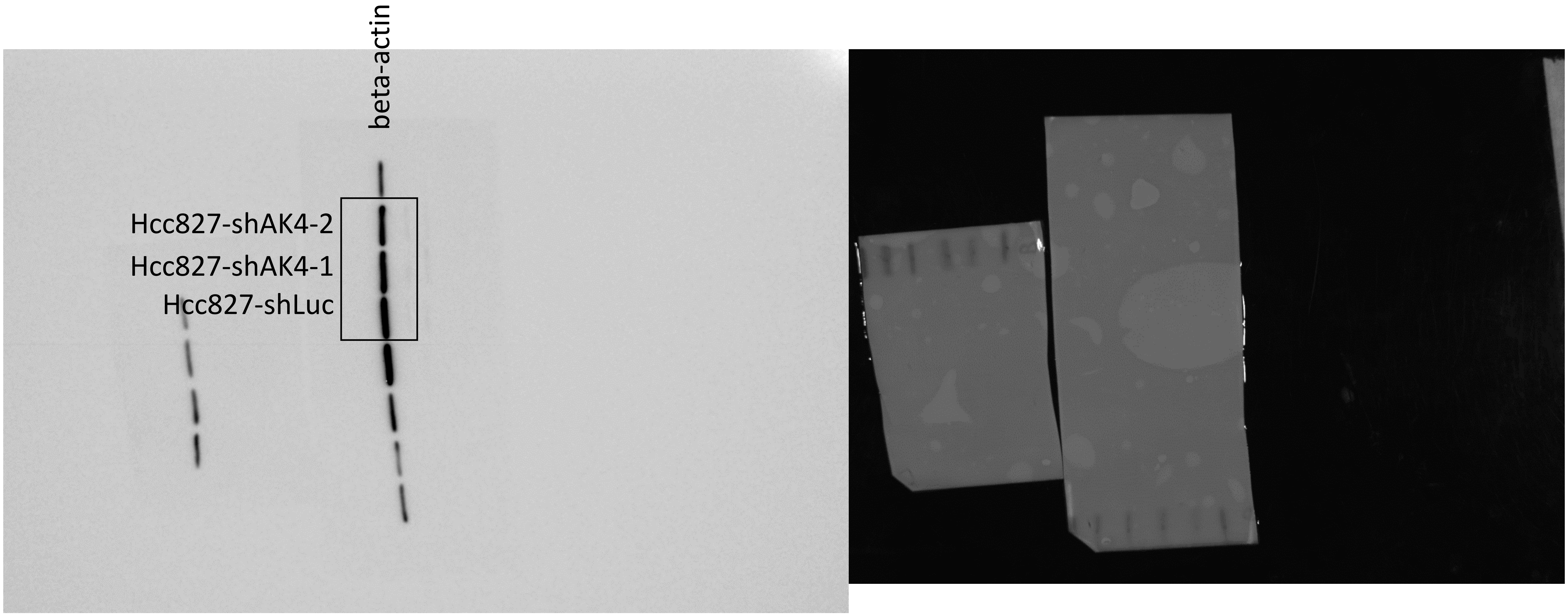

Supplement: Supplementary file 6 — raw data of blots [file 41598_2019_48243_MOESM6_ESM.pdf]
